# Insights into Immune Exhaustion in Chronic Hepatitis B: A Review of Checkpoint Receptor Expression

**DOI:** 10.3390/ph17070964

**Published:** 2024-07-21

**Authors:** João Panão Costa, Armando de Carvalho, Artur Paiva, Olga Borges

**Affiliations:** 1 Faculty of Pharmacy, University of Coimbra, 3000-548 Coimbra, Portugal; jpanao94@gmail.com; 2 CNC-UC—Center for Neuroscience and Cell Biology, University of Coimbra, 3004-504 Coimbra, Portugal; 3 CIBB—Center for Innovative Biomedicine and Biotechnology, University of Coimbra, 3004-504 Coimbra, Portugal; 4 Centro Hospitalar e Universitário de Coimbra, 3004-561 Coimbra, Portugal; aspcarvalho@gmail.com (A.d.C.); artur.paiva@chuc.min-saude.pt (A.P.); 5 Faculty of Medicine, University of Coimbra, 3004-504 Coimbra, Portugal

**Keywords:** chronic hepatitis B, immune checkpoint inhibitor, hepatitis B virus, co-inhibitory molecules, co-stimulatory molecules

## Abstract

Hepatitis B, caused by the hepatitis B virus (HBV), often progresses to chronic infection, leading to severe complications, such as cirrhosis, liver failure, and hepatocellular carcinoma. Chronic HBV infection is characterized by a complex interplay between the virus and the host immune system, resulting in immune cell exhaustion, a phenomenon commonly observed in chronic viral infections and cancer. This state of exhaustion involves elevated levels of inhibitory molecules, cells, and cell surface receptors, as opposed to stimulatory counterparts. This review aims to elucidate the expression patterns of various co-inhibitory and co-stimulatory receptors on immune cells isolated from chronic hepatitis B (CHB) patients. By analyzing existing data, the review conducts comparisons between CHB patients and healthy adults, explores the differences between HBV-specific and total T cells in CHB patients, and examines variations between intrahepatic and peripheral immune cells in CHB patients. Understanding the mechanisms underlying immune exhaustion in CHB is crucial for developing novel immunotherapeutic approaches. This detailed analysis sheds light on the immune exhaustion observed in CHB and lays the groundwork for future combined immunotherapy strategies aimed at leveraging checkpoint receptors to restore immune function and improve clinical outcomes.

## 1. Introduction

### 1.1. Hepatitis B Virus Infection: A Global Health Challenge in Need of Targeted Interventions

The Hepatitis B virus (HBV) can cause both acute and chronic liver disease, posing a potential life-threatening infection [1]. Despite the availability of an effective vaccine since the 1980s, the burden of chronic hepatitis B (CHB) remains substantial, with an estimated 254 million people living with hepatitis B in 2022 [2]. A cause for concern is that only 13% of people living with chronic hepatitis B infection had been diagnosed and close to 3% had received antiviral therapy at the end of 2022 [2]. Regions with the highest HBV prevalence include the WHO Western Pacific and African regions. The WHO African Region accounts for 63% of new hepatitis B infections, and yet only 18% of newborns in the region receive the hepatitis B birth-dose vaccination. The Western Pacific Region accounts for 47% of hepatitis B deaths, and treatment coverage remains low. HBV-related complications, such as cirrhosis, liver failure, and hepatocellular carcinoma, led to approximately 1.08 million deaths in 2022 [2]. Co-infection with other viruses, such as HIV (human immunodeficiency virus) and hepatitis C, further complicates the management of HBV infection [2]. Additionally, Hepatitis D virus (HDV) infection can occur as co-infection or superinfection with HBV, and it is the most severe form of viral hepatitis [3]. This infection is a significant complication of CHB, leading to a more severe form of liver disease and a higher rate of liver-related complications [3].

The global hepatitis epidemics have drawn the attention of international organizations, like the United Nations (UN). As part of the 2015–2030 Sustainable Development Goals, the UN is committed to combatting and eliminating viral hepatitis as a public health threat (Goal 3, target 3.2). Moreover, the UN supports research and development efforts for vaccines and medicines targeting communicable and non-communicable diseases that predominantly affect developing countries, including hepatitis B (Goal 3, target 3.B) [4]. The scale of the HBV problem requires targeted interventions and continued support from the global health community to effectively control and eradicate this infectious disease.

### 1.2. Hepatitis B Transmission and Strategies for Disease Eradication

Hepatitis B virus (HBV) transmission in endemic areas primarily occurs through perinatal transmission, with mother-to-child transmission during birth being a common route. Additionally, transmission can occur through percutaneous, mucocutaneous, or sexual routes. The outcome of HBV infection, whether acute or chronic, is influenced by the infecting viral dose and the age of infection [1]. Early acquisition of HBV infection is associated with a higher likelihood of chronicity, mainly due to the differences in immune maturity between adults and children [5]. Infants infected during the first year of life have a high chronicity rate of approximately 90%, while less than 5% of adults infected with HBV develop chronic hepatitis B (CHB). Of those with CHB, 20% to 30% may progress to cirrhosis and primary liver cancer [1].

To prevent the symptomatic disease and reduce liver cancer occurrence, HBV vaccination at birth is a critical intervention. The current hepatitis B immunization regimen includes a birth dose and two booster doses of a recombinant hepatitis B vaccine [5]. Remarkably, over 95% of vaccinated infants develop protective levels of anti-HBs antibodies after completing the full HBV vaccine series. The World Health Organization (WHO) strongly recommends administering the first hepatitis B vaccine dose to all infants as soon as possible, ideally within 24 h after birth, to control perinatal HBV infection [6]. Implementing vaccination strategies is crucial in the global efforts to eradicate HBV and significantly reduce the burden of CHB and its complications, like liver cancer.

### 1.3. Hepatitis B Virus: Pathogenesis, Replication, and Genotypes

Hepatitis B virus (HBV) is a hepatotropic DNA virus belonging to the *Hepadnaviridae* family. It is responsible for causing both acute and chronic liver disease, creating a significant global health burden. The HBV particle (virion) consists of an icosahedral nucleocapsid core composed of a core protein and containing the viral genome, surrounded by an envelope composed of lipids and surface proteins. The spherical infectious virions (known as “Dane particles”) have a mean diameter between 30 nm to 42 nm and harbor a partially double-stranded, relaxed circular DNA (rcDNA) genome of approximately 3200 bp. One end of the full length strand is linked to the DNA polymerase [7].

**Figure 1:**
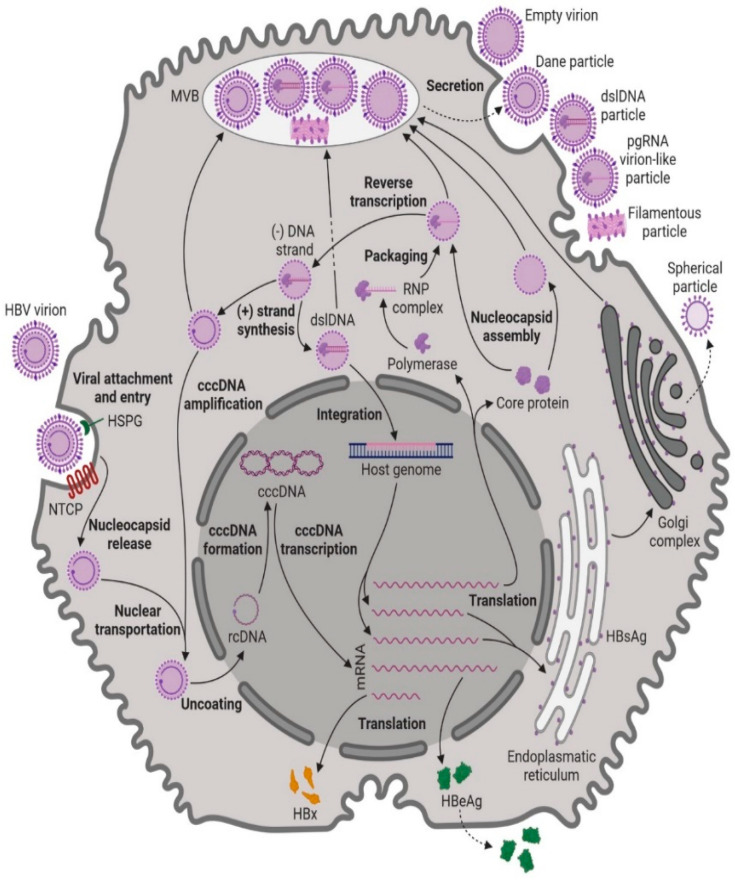
HBV replication cycle after entering the hepatocyte. Image created with BioRender.com.

The hepatitis B virus (HBV) genome consists of four genes, namely S, C, P, and X, which collectively encode a total of seven proteins. The S gene encodes the surface envelope glycoproteins, known as hepatitis B surface antigens (HBsAgs). The S gene is divided into three domains: preS1, preS2, and S [8]. The S gene contains three in-frame “start” codons (ATG), resulting in the generation of three different-sized proteins: protein S (S), protein M (preS2, S), and protein L (preS1, preS2, and S). Notably, protein L contains the outer domain preS1, which plays a crucial role in virion infectivity, as it is responsible for binding to receptors on the surface of hepatocytes [9]. The C gene encodes the core protein (HBcAg), which is responsible for forming the viral nucleocapsid. Additionally, the C gene encodes the pre-core protein, which undergoes proteolytic processing to yield the soluble antigen HBeAg. While HBeAg is not essential for viral replication, it is considered critical for the establishment of chronic HBV infection [7]. The P gene encodes the HBV DNA polymerase, which possesses reverse transcriptase activity and is essential for viral replication. Finally, the X gene encodes protein X, a transcription regulator that plays a role in modulating viral gene expression [9,10]. These various proteins encoded by the HBV genome play essential roles in the life cycle of the virus, replication, and infection, highlighting their significance in the development and progression of hepatitis B infection. The HBV infection process involves a series of intricate steps that facilitate viral entry, replication, and persistence within hepatocytes (Figure 1). Firstly, the virus attaches with low affinity to heparan sulphate proteoglycans (HSPGs) present on the surface of the hepatocyte. Subsequently, it connects with high affinity to sodium taurocholate co-transporting polypeptide (NTCP), a carrier protein situated in the basolateral membrane of the hepatocyte, enabling endocytic entry of the hepatitis viruses [11]. During translocation within the hepatocyte, the endosome’s pH decreases, leading to the fusion of the endosomal membrane with the viral envelope and the release of the nucleocapsid [12]. The viral nucleocapsids are then transported to the nucleus, where they disassemble, releasing the HBV relaxed circular DNA (rcDNA) [13]. The rcDNA is then converted into covalently closed circular DNA (cccDNA), a stable form of episomal viral DNA that is wrapped by host histones to form a mini-chromosome [8].

The cccDNA serves as a template for HBV replication and is transcribed into five different HBV RNAs of varying lengths: the 0.7 kb, 2.1 kb, 2.4 kb, and two types of 3.5 kb HBV RNAs, namely the pre-genomic RNA (pgRNA) and pre-core mRNA [9]. These viral RNAs are translocated to the cytoplasm, where they are translated into HBV proteins. The 0.7 kb RNA is translated into HBx, while the 2.1 kb RNA can be translated into both small (S) and middle (M) forms of the HBsAg. The 2.4 kb RNA is translated into the large (L) form of HBsAg. The 3.5 kb pre-core mRNA ultimately produces mature HBeAg, which is released into the bloodstream. Part of the 3.5 kb pgRNA is translated into the HBV DNA polymerase and core protein, while the other part is encapsulated into nucleocapsids to serve as a template for HBV replication [14].

After the formation of L, M, and S-HBsAg, they undergo processing in the endoplasmic reticulum (ER) and Golgi apparatus [14]. These processed HBsAg molecules can produce two types of capsid-free subviral particles: spherical particles, which mainly contain S-HBsAg and are secreted directly through the Golgi apparatus, and filamentous particles, which contain mostly S-HBsAg and identical amounts of L and M-HBsAg, and which are secreted by the hepatocyte via the multivesicular bodies (MVB) pathway [15].

The HBV DNA polymerase and pgRNA undergo structural alterations to form a P-ε ribonucleoprotein (RNP) complex. The RNP complex is encapsidated by core proteins, forming core particles [16]. Inside the core particles, reverse transcription occurs, and using the pgRNA as a template, HBV (−)-strand DNA is formed. The HBV DNA polymerase then accurately translocates to produce HBV (+)-strand DNA, resulting in nucleocapsids containing rcDNA [17]. These nucleocapsids can either be enveloped via the MVB pathway and later secreted as newly formed virions, along with other subviral particles, or be translocated to the nucleus and uncoated to replenish the cccDNA pool through an intracellular cccDNA amplification pathway [18].

Alternatively, an incorrect translocation of HBV DNA polymerase may lead to the synthesis of double-strand linear DNA (dslDNA), a form that can integrate into the host genome and serve as a template for HBsAg production [19]. Although unnecessary for virus replication, random cccDNA integration into the host genome ensures HBV persistence in the hepatocyte and provides an oncogenic potential [7].

The hepatitis B virus (HBV) displays significant genetic diversity, resulting in the classification of ten distinct genotypes (A to J) based on nucleotide sequence variations [20]. These genotypes display diverse distributions across various ethnic populations and geographic regions [21]. Genotypes B and C are predominantly found in East Asia, largely due to vertical transmission, while genotypes A and D are more prevalent in sub-Saharan Africa, where HBV infections are often transmitted horizontally [21]. In North America and Europe, genotypes A and D are the most relevant [22]. The HBV genotype plays a crucial role in influencing the viral pathogenesis and contributes to varying clinical outcomes and treatment responses among CHB patients [20]. For instance, patients infected with genotypes C and D have a lower frequency of spontaneous HBeAg seroconversion, which is a significant event in the natural course of the disease [20]. Moreover, compared to genotypes A and B, individuals with genotypes C and D face a higher risk of developing liver cirrhosis and hepatocellular carcinoma (HCC), the most severe complications of chronic HBV infection [22].

### 1.4. CHB Disease Course

The clinical course of CHB can vary significantly among patients, with some individuals progressing to severe liver complications, such as cirrhosis and HCC, while others maintain lifelong latent infections without requiring antiviral therapy [5]. 

CHB can be categorized into five disease phases according to the European Association for the Study of the Liver (EASL) (Table 1), which are not necessarily sequential, based on levels of HBV DNA, alanine aminotransferase (ALT), HBeAg status, and liver inflammation [10].

Phase 1 was previously termed the immune tolerant phase; it is prolonged and more commonly observed in infants infected after birth, due to normal HBV-specific T cell activity [23]. Patients in this phase are highly infectious, given the high values of HBV DNA [10]. After phase 1, the HBeAg-positive chronic hepatitis B phase is reached more rapidly in patients infected during adulthood. At the end of phase 2, HBeAg seroconversion usually occurs, with the appearance of anti-HBe antibodies and the consequent loss of HBeAg. Most subjects achieve HBV DNA suppression and transition to the HBeAg-negative chronic HBV infection phase (Phase 3), while others continue with the infection in the HBeAg-negative chronic hepatitis B phase for several years (Phase 4) [5,24]. During phase 3 (previously named the inactive carrier phase), some individuals may have HBV DNA levels between 2000 and 20,000 IU/mL, always with normal ALT values [10]. The risk of disease progression to cirrhosis and HCC is low if the patient remains in this phase. However, CHB may develop, leading to typical complications. Per year, 1% to 3% of patients can achieve HBsAg seroconversion and/or HBsAg loss, considered a functional HBV infection cure, with a low rate of intrahepatic viral replication [25]. Throughout phase 5, in cases of HBsAg loss before cirrhosis onset, the risk of developing cirrhosis and cancer is slight, with a significant improvement in survival rates. However, HBV reactivation may occur in immunosuppressed patients [10].

### 1.5. Treatment

The primary therapeutic objective in hepatitis B virus (HBV) infection is to enhance the quality of life and survival while preventing disease complications and progression, such as cirrhosis and HCC development [10]. The ultimate goal of treatment plans is achieving sustained suppression of HBV replication, with the optimal endpoint being the loss of HBsAg [10].

Currently, oral nucleos(t)ide analogue therapy is the mainstay of HBV infection treatment globally. First-line choices include entecavir, tenofovir alafenamide, and tenofovir disoproxil fumarate, which predictably induce high HBV suppression, leading to undetectable levels of serum HBV DNA in over 95% of compliant patients [5]. These drugs offer a significant advantage as they are the only treatment option for various patient subgroups, such as liver transplant recipients, individuals with acute hepatitis B, severe chronic HBV exacerbation, and immunosuppressed patients requiring HBV reactivation prevention [26]. Furthermore, oral nucleos(t)ide analogue therapy is considered safe, with a favorable tolerability profile and a low incidence of viral advances due to the high barrier to HBV resistance [27]. However, despite effectively controlling new virion production and viremia, these drugs cannot eliminate cccDNA persistence or prevent HBsAg and HBeAg expression and release [9]. Long-term use of nucleos(t)ide analogues can lead to substantial benefits in patient recovery [28]. Treatment for more than 5 years is associated with reduced intrahepatic cccDNA levels, decreased incidence of complications, and meaningful histological improvement, including cirrhosis regression [29,30,31]. The success of entecavir, tenofovir alafenamide, and tenofovir disoproxil fumarate has reduced the use of the older-generation nucleos(t)ide analogues, such as adefovir, telbivudine, and lamivudine [5].

Another first-line therapy for CHB patients is the pegylated interferon alfa, administered via subcutaneous injection for a maximum period of 1 year [5]. This CHB treatment possesses immune modulatory and direct antiviral properties, but it is associated with significant adverse effects and variable patient responses [9,10]. Additionally, the low rate of sustained viral suppression after therapy discontinuation and numerous contraindications compared to oral nucleos(t)ide analogues make pegylated interferon a less popular alternative [5,10]. While theoretically, combined therapy of pegylated interferon and nucleos(t)ide analogues may be advantageous, the evidence for this approach is still lacking [10,32].

Patient response to nucleos(t)ide analogues is generally similar across different HBV genotypes, with patients infected with genotypes A and B responding better to interferon alfa therapy than those with genotypes C and D [21].

As of now, HBV infections remain difficult to cure, primarily due to the integration of HBV cccDNA into the patient’s genome [8,10]. This integration makes it extremely challenging to completely eliminate cccDNA and achieve viral eradication, thereby making cccDNA eradication the ultimate goal of antiviral treatment for chronic HBV patients [8].

Consequently, new therapies or combinations of existing treatments are needed to achieve complete eradication of the virus. To this end, it is essential to better understand the circumstances under which the immune system can effectively combat the infection and what factors contribute to its failure in other situations. The following review aims to provide a comprehensive overview of what is currently known about the characteristics of immune system cells in the context of chronic hepatitis B disease.

## 2. HBV and the Immune System

### 2.1. HBV Infection Natural Control

In acute HBV infection, the immune response relies on both innate and adaptive immunity to effectively control the viral invasion. An effective immune response involves the production of anti-HBV antibodies, the secretion of cytokines, and the generation of HBV-specific CD8^+^ and CD4^+^ T cells [33]. 

The innate immune cell components play a crucial role in detecting the presence of the virus through recognition of viral proteins, nucleic acids, and tissue damage, if present [34]. Their main function is to reduce viral replication and antigen levels until the adaptive immune response is fully activated [33]. During self-limited infections, there is a significant decline in viral DNA, reaching a reduction of 90% within 2–3 weeks after the peak of HBV replication [34]. This decline occurs before the onset of liver cell destruction, which is achieved through non-cytopathic processes involving not only T cells [35] but also, and equally importantly, natural killer (NK) cells, with both secreting antiviral cytokines, like IFN-γ and TNF-α, that promote the activation and support the maturation and recruitment of adaptive immune components [33,36].

The adaptive immune response involves the maturation and expansion of B and T cells, which can specifically detect and target cells with the infectious agents. In the liver, the immune environment promotes a Th1 immune response, assisting T cells in eliminating infected liver cells and effectively controlling HBV infection [36]. Moreover, the adaptive immune response induces the development of memory T cells, which provide protective properties against future infections [34]. This memory response is essential for long-term immunity and contributes to the ability of the immune system to combat future HBV exposures [37].

However, in cases where the infected patient fails to generate an adequate antiviral immune response, the infection is likely to persist and become chronic [33]. Chronic HBV infection occurs when HBV manages to evade clearance by the immune mechanisms during the acute phase, leading to a dormant state where the virus does not cause immediate disease or organ damage [38].

### 2.2. HBV Persistency

Several pathogens, including HBV, have evolved strategies to escape the host’s immune response and establish chronic infections. The persistence of chronic HBV (CHB) infection involves a complex interaction between the virus and the host’s immune system, leading to the exhaustion of both innate and adaptive immune responses [35].

The age at which the individual acquires the HBV infection plays a significant role in determining the likelihood of chronicity. Perinatal infections are more likely to lead to HBV persistence, while infections in adults can often be controlled [5,24]. In the case of perinatal infection, another important aspect is the mother’s state of infection. Maternal HBeAg production during perinatal transmission can also contribute to HBV persistence in the newborn, leading to elevated levels of HBV DNA, HBsAg, and HBeAg, along with the tolerogenic hepatic environment, promoting various immune exhaustion mechanisms [39].

HBV has developed mechanisms to evade the innate immune system’s recognition and antiviral activities, thereby prolonging the infection. The covalently closed circular DNA (cccDNA), present in the nucleus of infected cells, avoids recognition by innate DNA-sensing cellular machinery [34]. Additionally, cccDNA produces viral mRNA that resembles typical cellular transcripts, further suppressing the immune response [40]. In the end, these tactics lead to reduced HBV sensing and a lack of type I interferon (IFN) secretion [40]. 

Intrinsic antiviral immunity is a specialized component of innate immunity that directly obstructs viral replication and assembly, thereby rendering cells resistant to specific viruses [41]. This form of immunity is mediated by restriction factors that are typically preexistent in certain cell types, although they can be further induced in response to viral infection. These intrinsic virus-restriction factors identify specific viral components and inhibit viral replication immediately and directly, distinguishing them from other pattern-recognition receptors that act indirectly by inducing interferons and other antiviral molecules. However, some viruses have evolved mechanisms to evade this intrinsic antiviral defense and successfully replicate within host cells. For example, the structural maintenance of the chromosome 5/6 complex (Smc5/6) is a restriction factor that represses the transcription of HBV cccDNA, when associated with Nuclear Domain 10 (ND10) [42]. HBV circumvents this restriction by producing the HBV X protein (HBx), which targets Smc5/6 for degradation and thereby enhances HBV gene expression [42]. The detailed mechanism by which Smc5/6 suppresses HBV transcription and the initial expression pathway of HBx remain unknown.

The activity of various HBV proteins can also contribute to immune suppression. HBV proteins disturb the cellular production of antiviral cytokines, like type I IFNs, by interfering with the usual intracellular signaling during HBV infection [34]. For instance, the HBV polymerase interferes with interferon regulatory factor (IRF) to inhibit IFN-β production, a key antiviral protein involved in the innate immune response [43]. The HBV X protein can interfere with cytosolic sensory molecules responsible for inducing the release of type I IFN [44,45]. Moreover, HBV antigens, such as HBsAg and HBeAg, actively secreted during viral replication, can suppress Toll-like receptor (TLR)-induced immune responses [46].

### 2.3. Immune Cell Exhaustion

**Figure 2:**
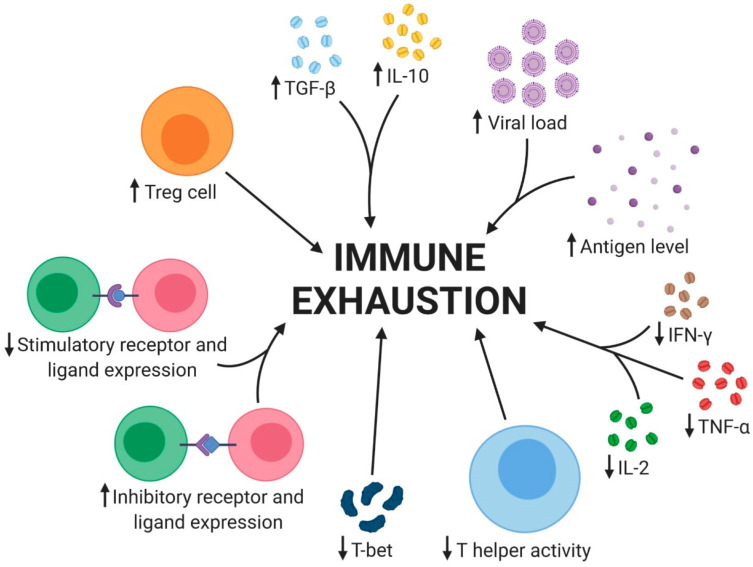
Mechanism and key factors leading to immune cell exhaustion in a CHB scenario. The increased HBV viral load and antigen level overwhelm the immune system, while cytokine imbalance, increased inhibitory receptors and ligands, decreased stimulatory receptors and ligands, increased regulatory T cells, decreased T helper activity, and decreased T-bet expression all contribute to T-cell exhaustion. Image created with BioRender.com.

Immune cell exhaustion is a complex process involving various immunoregulatory mechanisms. It can be categorized into three major groups: soluble factors, cell types, and cell surface receptors [47]. In the state of exhaustion, there is an increase in suppressive cells and molecules, while stimulatory features are often diminished (Figure 2). These suppressive cells and molecules can actively promote immune exhaustion or be influenced by the suppressive microenvironment. In CHB, immune exhaustion is characterized by several key factors. The increased HBV viral load and antigen level overwhelm the immune system, leading to T-cell exhaustion. This is accompanied by a cytokine imbalance, with increased levels of transforming growth factor-beta (TGF-β) and interleukin-10 (IL-10), which suppress the immune response. In contrast, the levels of interleukin-2 (IL-2), tumor necrosis factor-alpha (TNF-α), and interferon-gamma (IFN-γ) decrease, further contributing to immune exhaustion. The expression of inhibitory receptors and ligands is increased, suppressing T-cell activation and function. Conversely, the expression of stimulatory receptors and ligands is decreased, further contributing to T-cell exhaustion. The immune response is also impaired by the increased expression of regulatory T cells (Tregs) and the decreased activity of T helper cells. Tregs suppress the immune response, while decreased T helper activity impairs the ability to mount an effective response against the virus. Additionally, the decreased expression of T-bet, a transcription factor essential for T-cell activation and differentiation, further contributes to T-cell exhaustion. These factors collectively lead to the immune exhaustion observed in CHB, making it challenging for the immune system to effectively control the virus and leading to the development of chronic liver disease.

**Figure 3:**
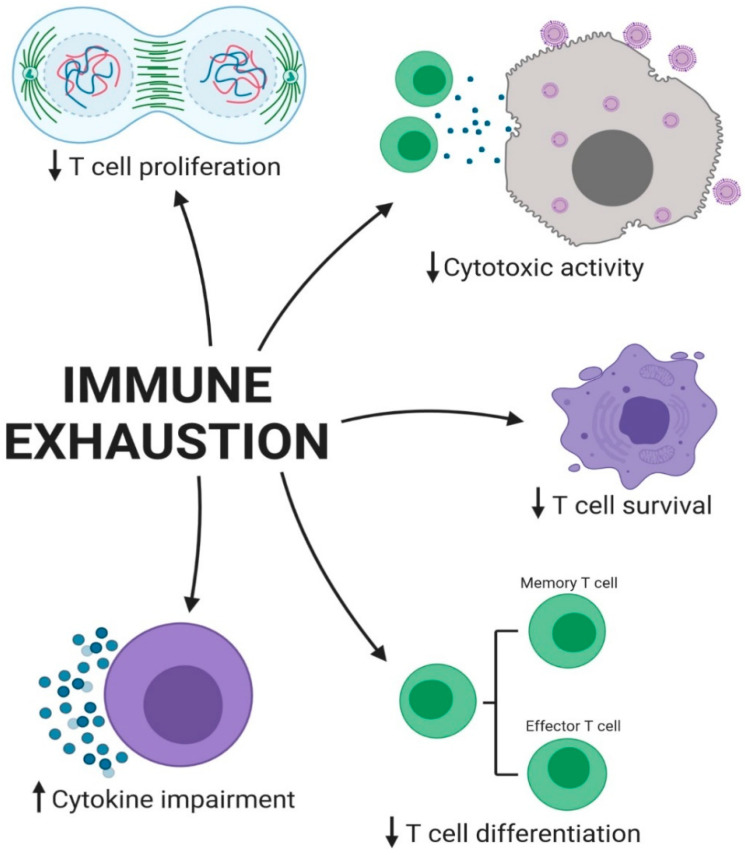
Outcomes of immune cell exhaustion in CHB patients. Immune cell exhaustion in CHB patients leads to decreased T cell proliferation, survival, and differentiation, as well as impaired cytokine responses and decreased cytotoxic activity. This exhaustion results in a lack of effective T cell responses against the virus, making it challenging for the immune system to effectively clear the infection.

Immune cell exhaustion in CHB patients leads to several outcomes that impair the immune response and contribute to the persistence of the infection (Figure 3). One of the key outcomes is decreased T cell proliferation, making it challenging for the immune system to effectively respond to the virus. Additionally, the exhausted T cells have a shorter lifespan, leading to a decrease in the overall number of functional T cells available to combat the infection. Furthermore, the T cells in CHB patients are unable to differentiate properly, resulting in a lack of effective T cell responses against the virus. This impaired differentiation is a critical aspect of immune exhaustion, as it prevents the immune system from mounting an effective response against the infection. The cytokine imbalance in CHB patients also leads to impaired immune responses, making it difficult for the immune system to effectively clear the virus Finally, the cytotoxic activity of T cells is decreased, making it challenging for the immune system to effectively eliminate infected cells and control infection. This decreased cytotoxic activity is a critical aspect of immune exhaustion, as it prevents the immune system from effectively clearing the virus and controlling the infection. Overall, the outcomes of immune cell exhaustion in CHB patients highlight the complex interplay between the immune system and the virus, as well as the critical role that immune exhaustion plays in the persistence of the infection.

#### 2.3.1. Soluble Factors

Soluble factors, such as certain immunoregulatory cytokines and chemokines, play a crucial role in immune regulation and exhaustion. Among these, IL-10 stands out as a predominantly immunosuppressive cytokine capable of reducing inflammatory responses. IL-10 exerts inhibitory effects on CD4^+^ and CD8^+^ T cell proliferation and cytokine production, as well as altering the function of antigen-presenting cells (APCs), such as B cells, dendritic cells (DCs), and macrophages [48]. Various cell types, including T cells, DCs, B cells, macrophages, and Treg cells, are capable of producing IL-10, and its production can be induced by IL-10 (positive feedback), IL-27, and TGF-β [49]. Elevated levels of IL-10 are commonly observed in CHB viral infections and are associated with T cell impairment, as neutralizing IL-10 can boost T cell function and improve viral control [50]. Thus, high levels of IL-10 are a hallmark of many chronic infections.

TGF-β is another immunosuppressive cytokine that is implicated in T cell dysfunction. It plays a crucial role in controlling the differentiation, proliferation, adhesion, and survival of various cell types and is essential in suppressing autoreactive T cells [51]. TGF-β signaling is responsible for inducing apoptosis in virus-specific CD8^+^ T cells. However, when TGF-β signaling is selectively attenuated, T cell numbers increase, and their functions are restored, leading to viral clearance and the generation of memory immunity [52].

In contrast to immunosuppressive cytokines, some cytokines act as positive regulators, enhancing immune responses during chronic infections [47]. Treatment with IL-2 or IL-7 in mice with chronic viral infections has been shown to improve the immune response of impaired T cells [53,54]. Additionally, IL-21 plays a critical role in persistent viral infections. In chronic infections, virus-specific CD4^+^ T cells produce IL-21, which is essential for CD8^+^ T cells to avoid deletion, sustain immunity, and resolve the persistent infection [55]. 

Thus, in chronic infections, the quality and efficiency of the immune response can be significantly influenced by changes in both positive and negative regulatory cytokines [47].

#### 2.3.2. Cell Types

Various cell types play key roles in dampening immune responses and promoting immune tolerance, which can have significant implications in chronic infections. By reducing the suppressive influence of these cells, it may be possible to restore and enhance immune responses, leading to better control of chronic infections and improved outcomes in immunotherapy. 

##### NK Cells

NK cells play a pivotal role in the innate immune response against viral infections, including HBV. Their ability to recognize and eliminate infected cells without the need for specific antigen recognition, as seen in adaptive immunity, makes them essential in the early control of viral replication [56]. NK cells represent around 30% to 40% of intrahepatic lymphocytes and they are able to recognize and eliminate HBV infected cells, depending on the balance between negative and positive signals [34].

The activation of NK cells in response to viral infections is largely mediated by cytokines such as type I interferons (IFNs) and IL-12, which promote their effector functions [57]. Once activated, NK cells release IFN-γ, which not only directly inhibits viral replication but also helps in shaping the adaptive immune response. IFN-γ stimulates CD4^+^ T cells, promoting a Th1 polarization that is important for effective antiviral immunity [58].

However, in the context of chronic HBV infection, the immunosuppressive environment created by elevated levels of inhibitory cytokines, such as IL-10 and TGF-β, can impair NK cell function [59]. These cytokines can inhibit IFN-γ production by NK cells, compromising their antiviral activity. Moreover, NK cells from CHB patients may themselves produce higher levels of anti-inflammatory cytokines and express more inhibitory receptors, such as PD-1, which further contribute to their suppressive state [60]. This suppressive state of CHB infection also leads to NK cell differentiation into regulatory NK cells (NKreg cells) that can directly dampen the antiviral activity of NK cells and also inhibit HBV-specific T cell responses, contributing to immune evasion and viral persistence [61].

##### Monocytes and Macrophages

Monocytes and macrophages are essential components of the innate immune system and play a crucial role in detecting and responding to pathogens, including HBV [62]. They act as professional phagocytes, engulfing and eliminating invading pathogens and infected cells. However, in the context of HBV infection, these immune cells can undergo changes in their functional profile, leading to immunosuppression and impaired antiviral responses [56]. HBV has been shown to induce a suppressive profile in monocytes and macrophages, characterized by alterations in cytokine secretion and up-regulation of inhibitory molecules. This suppressive profile includes higher secretion of immunosuppressive cytokines, such as TGF-β and IL-10, which can inhibit the activation of other immune cells and dampen the overall immune response. At the same time, there is a decrease in the production of pro-inflammatory cytokines, such as TNF-α and IL-12, which are essential for promoting antiviral immunity. The upregulation of inhibitory molecules, like PD-L1, is also a key mechanism used by HBV to evade the immune system [60,61,63].

##### Myeloid Derived Suppressive Cells

Myeloid-derived suppressive cells (MDSCs) are typically absent in healthy individuals but emerge predominantly in cancer and pathological conditions associated with chronic inflammation and stress [64]. Derived from bone marrow precursors and closely related to neutrophils and monocytes, MDSCs exert their suppressive effects primarily on T cells, as well as on NK cells, DCs, and macrophages [65]. The main mediators of MDSC-mediated suppression include TGF-β, IL-10, arginase, and indoleamine 2,3-dioxygenase (IDO) [64]. By producing arginase, MDSCs interfere with T cell responses, leading to reduced IFN-γ secretion by antigen-specific T cells [66]. Additionally, IDO-driven tryptophan catabolism hampers T cell and NK cell proliferation, while also generating specific ligands that modulate lymphocyte functions [67]. The depletion of vital amino acids by MDSCs further inhibits lymphocyte anabolic functions, contributing to the establishment of an immunosuppressive microenvironment [56]. In addition, MDSCs can induce regulatory T cell (Treg) formation and influence reactive oxygen species (ROS) production [60]. Consequently, MDSCs play a critical role in the development and persistence of CHB infection [68]. CHB patients demonstrate higher MDSC levels compared to healthy adults, with MDSC percentages being directly correlated with the viremia state in HBV-infected patients [69]. 

##### Dendritic Cells

Dendritic cells (DCs) play a central role in initiating and modulating immune responses by presenting antigens to T cells and producing cytokines that determine T cell polarization [60]. In chronic hepatitis B (CHB), DCs are significantly impacted, leading to altered immune regulation and compromised antiviral immunity. DCs can be categorized into two main subsets: conventional or myeloid DCs (mDCs) and nonclassical DCs, including plasmacytoid DCs (pDCs), monocyte-derived DCs (MoDCs), and inflammatory monocyte-derived DCs (infDCs) [70]. 

The percentage of mDCs is reduced in CHB patients, and this decline is inversely correlated with the plasma HBV load. Moreover, mDCs from CHB patients exhibit elevated expression of programmed cell death ligand-1 (PD-L1) with concomitant high levels of alanine transaminase (ALT). Additionally, pDCs in CHB patients demonstrate impaired production of IFN-α, further complicating antiviral immune responses [71]. MoDCs from HBV-infected patients exhibit compromised cytokine secretion, particularly a reduction in IL-12 production. This deficiency in IL-12 leads to decreased TNF-α and IFN-γ production by T cells, impairing the host’s capacity to mount effective antiviral responses [72].

##### Regulatory T Cells

Regulatory T cells (Treg cells) are a critical subset of CD4^+^ T cells expressing the transcription factor FoxP3, essential for immune regulation and self-tolerance [60]. They can be classified into two subtypes: “natural” Treg cells (nTreg cells), originating in the thymus, and “induced” Treg cells (iTreg cells), recognized as key immune resistance mediators [56].

Overexpression of FoxP3, potentially induced by transforming growth factor-beta (TGF-β), confers suppressive activity to conventional T cells, converting them into Treg cells [49]. Treg cells exert immunosuppression by releasing inhibitory cytokines (TGF-β, IL-10, IL-35) and upregulating inhibitory surface receptors (PD-1, CTLA-4, Tim-3, LAG-3), leading to T cell effector function limitation, including IFN-γ secretion inhibition [73].

In chronic hepatitis B (CHB) patients, both peripheral and intrahepatic Treg cell frequencies are elevated compared to healthy adults, with a positive correlation between Treg cell level and plasma HBV load [74,75,76,77]. This suggests that Treg cells directly and indirectly dampen T cell immune responses, potentially promoting HBV infection persistence [36,60].

##### T Cells

The adaptive immunity, particularly the HBV-specific CD4^+^ and CD8^+^ T cell response, plays a critical role in viral clearance and the immune pathogenesis of HBV [74,75,76]. 

CD4^+^ helper T cells recognize antigenic peptides presented by MHC class II molecules and produce cytokines, regulating effector and inflammatory immune responses, and facilitating CD8^+^ T cell activation and B cell antibody production [56]. Thus, the type of immune response elicited is defined by the pattern of cytokine produced and depends on the specific pathogen causing infection. The predominant immune response in HBV infection is Th1-based, characterized by IFN-γ production, leading to macrophage and CD8^+^ T cell activation and memory immunity formation [49]. Effector CD8^+^ T cells directly destroy target cells expressing antigens presented by MHC class I molecules [56]. Additionally, cytokines, like IFN-γ and TNF-α, are released by T cells and are crucial in reducing viral replication [77].

However, similar to innate immune cells, adaptive immune cells are restrained in CHB infection. A competent immune response against the virus fails to be mounted, resulting in a complex battle between immune tolerance and viral clearance, ultimately leading to immune cell exhaustion [75]. Continuously elevated serum HBV DNA, persistent exposure to high levels of HBsAg and HBeAg, and tolerogenic features of liver cells cause T cell tolerance and virus-specific T cell immune exhaustion [35,60]. Suppressive molecules (IL-10, TGF-β, arginase) and the influence of MDSCs, NK reg cells, and Treg cells further promote T cell dysfunction [73].

T cell exhaustion is characterized by the dysfunction and subsequent deletion of total and antigen-specific T cells during chronic viral infections and cancer [47,78]. CD8^+^ T cells lose effector function due to persistent stimulation, leading to immunopathologic damage and viral persistence [79]. Exhausted T cells were identified for the first time in a chronic lymphocytic choriomeningitis (LCMV) infection as virus-specific CD8^+^ T cells that did not secrete cytokines [78]. Loss of function happens in a hierarchical and gradual manner during exhaustion, with impaired CD8^+^ T cells losing their properties progressively throughout different stages [80]. Firstly, CD8^+^ T cells begin to lose some functions, like their proliferative capacity and ability to produce IL-2. In intermediate stages, other properties are lost, such as the production of TNF-α. Severe dysfunction leads HBV-specific T cells to partially or completely lose the ability to, for instance, secrete large amounts of IFN-γ. In the last stages of exhaustion, virus-specific T cells end up being physically deleted [81]. Moreover, a high viral load and long-term infection lead to increased expression of inhibitory surface receptors on T cells [47]. 

#### 2.3.3. Cell Surface Receptors

T cell responses are tightly regulated by co-stimulatory and co-inhibitory interactions, crucial for maintaining immune homeostasis and preventing autoimmunity. Immune checkpoint receptors play a vital role in balancing immune responses by providing inhibitory signals. Notably, Professor Tasuku Honjo and Professor James Allison were recognized with the 2018 Nobel Prize in Physiology or Medicine for their groundbreaking work on PD-1 and CTLA-4, respectively, in cancer research and treatment [82]. In physiological conditions, inhibitory receptors ensure self-tolerance and control excessive inflammation during infection diseases [83]. Conversely, co-stimulatory receptors activate immune cells, eliciting an efficient response against pathogens. The initiation of T cell response occurs when the T cell receptor (TCR) interacts with antigen-presenting cells (APCs) displaying an antigen–major histocompatibility complex (MHC). The subsequent ligand–receptor interactions between APCs and T cells regulate the nature of the T cell response, whether inhibitory or stimulatory [56]. These regulatory processes occur at the lymph nodes during T cell activation or in peripheral tissues and tumors.

**Figure 4:**
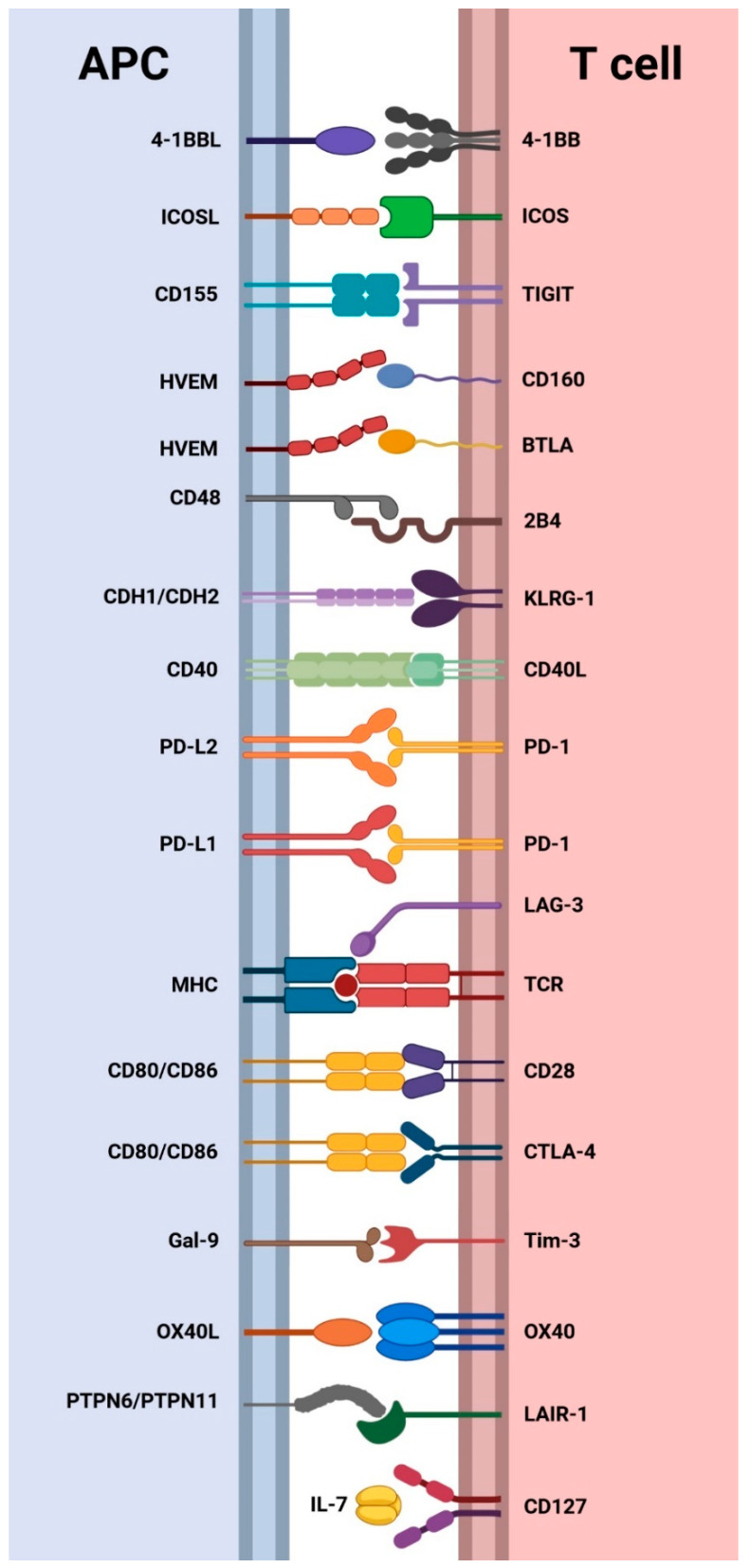
Depiction of co-stimulatory and co-inhibitory ligand–receptor interactions between APCs and T cells studied in CHB patients. The regulatory molecules and their ligands are depicted, highlighting the key interactions that influence T cell function. Image created with BioRender.com.

For T cells to respond to ligand–receptor interactions, antigen recognition by TCR is essential. In some cases, the same ligand can bind to pairs of co-stimulatory and co-inhibitory receptors, exemplified by CD80 or CD86 binding to CD28 or CTLA-4, respectively, resulting in positive or negative regulation of T cell responses (Figure 4) [84].

The communication between APCs and T cells can be bidirectional, as seen in CD40/CD40L interactions, where the activated T cell overexpresses the ligand, engaging with its receptor on APCs and inducing a cellular response [85]. 

The sustained and elevated expression of immune checkpoints is a key factor contributing to CD4^+^ and CD8^+^ T cell exhaustion, despite the transient expression of inhibitory receptors during T cell activation [86]. This prolonged upregulation of co-inhibitory signals is coupled with the downregulation of co-stimulatory receptors, leading to enhanced T cell dysfunction [87]. Figure 4 illustrates the co-stimulatory and co-inhibitory ligand–receptor interactions between APCs and T cells studied in CHB patients. The next section of this review aims to analyze the scientific literature concerning the expression of cell surface co-inhibitory receptors in CHB.

## 3. Expression of Cell Surface Co-Inhibitory Receptors in CHB

A comprehensive literature review was conducted to investigate the extent and diversity of immune checkpoint receptors involved in the context of CHB infection. The primary objective was to analyze whether immune cells from CHB patients exhibit a more pronounced exhaustion profile compared to healthy adults. Moreover, the study sought to identify phenotypic differences between peripheral and intrahepatic T cells, as well as between total and HBV-specific T cells. The ultimate goal of this analysis is to facilitate informed decisions regarding potential antibody treatments for the restoration of CHB patient immunity.

The selected papers were organized into tables based on the investigated receptor/ligand. Only studies that focused on chronic hepatitis B patients, explored relevant immune cell subsets, and were written in English were included. Immune cells were isolated either from peripheral blood or hepatic tissue obtained from liver biopsy. It is important to note that the patient pool exhibited variability in terms of disease phase, HBV genotype, and HBV treatment. Furthermore, some studies analyzed HBV-specific cells with varying epitope recognition between different research works. Additionally, certain studies expressed receptor frequency within cell populations in terms of percentage, while others used the number of positive cells per volume or determined mean fluorescence intensity (MFI).

### 3.1. CTLA-4, CD28, CD80, and CD86

CTLA-4 (cytotoxic T-lymphocyte-associated antigen 4) is a critical immune checkpoint receptor found on the surface of T cells that plays a key role in regulating T cell activation as it stops potentially autoreactive T cells at the initial stage of naive T-cell activation [56]. 

CTLA-4 is a CD28 homolog and functions as a counterbalance to CD28, a co-stimulatory receptor on T cells. Central to this process is the recognition and binding of a TCR to an antigen presented by the MHC on the surface of an APC. CD28 amplifies T cell activation upon initial antigen recognition by the TCR [88]. After antigen recognition, CTLA-4-mediated immune checkpoint is induced, and the levels of CTLA-4 depend on the amplitude of TCR signaling. Ligands with a high affinity for TCR induce higher levels of CTLA-4, leading to a stronger inhibition of the initial T cell response. Both CTLA-4 and CD28 bind to the same ligands on antigen-presenting cells (APCs), namely CD80 (B7.1) and CD86 (B7.2). However, CTLA-4 has a higher affinity for these ligands than CD28, thereby hampering T cell activation by outcompeting CD28 for their ligands and sending inhibitory signals to the T cell [89,90], such as decreased IL-2 production, reduced cell proliferation and survival [91]. Additionally, CTLA-4 receptors can sequester B7-ligands at the surface of APCs, resulting in the depletion of these ligands [92]. Consequently, CTLA-4 functions as a signal dampener to sustain T cell activation at steady levels, preventing excessive immune responses.

CTLA-4 plays a major role in downregulating helper T cells and enhancing Treg cells, although also being expressed by CD8^+^ T cells. Treg cells constitutively express CTLA-4 due to higher levels of Forkhead box P3 (FoxP3), a nuclear transcription factor that regulates CTLA-4 expression [92]. In contrast, naive and memory T cells have high levels of CD28 on their surface, while CTLA-4 is stored in intracellular vesicles. Only upon TCR binding to the antigen is CTLA-4 transported to the cell surface [56]. This immune checkpoint has attracted significant attention in the field of immunotherapy, and its blockade with anti-CTLA-4 antibodies shows potential as a treatment strategy for various diseases [93]. 

To better understand the mechanisms of immune cell exhaustion and identify potential targets for immunotherapeutic interventions, it is essential to characterize the immune cells of CHB patients, including the extent and specific cell types expressing co-inhibitory receptors. Blocking these inhibitory receptors using immune checkpoint inhibitors (ICIs) has shown promise as a potential therapeutic strategy for CHB. 

CTLA-4 is one of several co-inhibitory surface molecules that modulate T cell activation by blocking co-stimulation via CD28 and transmitting inhibitory signals to lymphocytes. As previously mentioned, the receptors CTLA-4 and CD28 on T cells compete for binding with CD80 and CD86, present on antigen-presenting cells (APCs). Consequently, studies on the expression of the cell receptors CTLA-4 and CD28 have primarily focused on Treg, CD4^+^, and CD8^+^ T lymphocytes, while investigations of CD80 and CD86 receptors have concentrated on dendritic cells (DCs), Kupffer cells (KCs), and B cells (Table S1—Supplementary Materials).

The expression of CTLA-4 on peripheral total CD4^+^ and CD8^+^ T cells appears to be low in both CHB subjects and healthy adults (HA) [23,94]. In contrast, studies on HBV-specific T cells from CHB patients reported a much higher frequency of CTLA-4-expressing cells within this T cell subgroup [95,96,97]. Indeed, the papers included in Table S1 indicate that HBV-specific CD4^+^ and CD8^+^ T cells of CHB patients might be particularly affected by immune exhaustion. Fisicaro et al. conducted a study comparing the expression of the co-inhibitory receptor CTLA-4 on HBV-specific CD8^+^ T cells isolated from two different compartments in CHB patients: intrahepatic and peripheral blood [96]. Their findings revealed that the expression levels of CTLA-4 were not significantly different between HBV-specific CD8^+^ T cells obtained from the liver and those obtained from the peripheral blood. This suggests that the upregulation of CTLA-4 on HBV-specific CD8^+^ T cells is not specific to the liver microenvironment, but rather a general characteristic of these exhausted T cells in CHB patients, regardless of their location. This is in contrast to Wang et al., who reported a higher frequency of CTLA-4^+^ cells among total CD8^+^ T cells in the liver compared to peripheral blood [98]. Tregs, especially those found in the hepatic tissue, express meaningful levels of CTLA-4 [75,96]. This means that these Tregs have a significant amount of CTLA-4 on their surface, which can interact with its ligand, CD80/CD86, on APCs.

The number of CD28^+^ T cells is generally not significantly different between CHB patients and healthy adults (HA), in both CD4^+^ and CD8^+^ T cells [94,99,100]. Although there is a lack of studies specifically focusing on HBV-specific T cells, the results on total T cells suggest that the exhaustion mechanism affecting CHB patients’ T cells does not particularly influence co-stimulation via CD28 expression. It is possible that T cell exhaustion can be induced by CTLA-4, which has a stronger affinity for CD80 and CD86 than CD28, without requiring alterations in CD28 expression. Interestingly, in the case of invariant natural killer T (iNKT) cells, the expression of CD28 was found to be much lower in CHB subjects than HAs [101]. 

On the other side of the immunological synapse, APCs express the co-stimulatory molecules CD80 and CD86, which are ligands for CTLA-4 and CD28. CD80 expression on APCs is associated with Th1 cell differentiation, while CD86 expression drives Th cells toward a Th2 profile [102].Therefore, downregulation of CD80 might result in the absence of a proper Th1 response required for HBV clearance, which could explain the lack of efficient HBV-specific T cells in CHB patients.

The immune cell subset mainly focused on CD80 and CD86 expression studies is dendritic cells (DCs), given their importance as APCs. All studies concerning DCs in general found a reasonable CD80 expression, although it was consistently lower in CHB patients (CHBp) than in healthy adults (HAs) [103,104,105,106,107]. However, when the authors specifically studied myeloid DCs (mDCs), the obtained results became more inconclusive. Nonetheless, Tjwa et al. reported a higher CD80 expression in CHB individuals than in HAs [108]. Plasmacytoid DCs (pDCs), liver Kupffer cells (KCs), and B cells seem to have an identical expression of CD80 between CHBp and HAs [102,109,110], whereas the percentage of CD80^+^ monocyte-derived DCs (MoDCs) is reduced in CHBp when compared with HAs [111].

Regarding CD86, studies in DCs generally found the number of CD86^+^ cells to be similar or lower in CHBp than in HAs [103,104,106,112,113]. CD86 expression on mDCs was similar to the expression of CD80; some studies found the frequency of CD86^+^ mDCs in CHBp to be higher, similar, or even lower than in HAs [108,109,111,114,115]. The pDCs expressed equivalent levels of CD86 in both subject groups, except for the results reported by Martinet et al. [109,115,116,117]. Unlike Chen et al., who found a significantly higher expression of CD86 in MoDCs from HAs [111], Said et al. reported a lower expression in KCs from HAs relative to CHBp [102]. Lastly, the studies that focused on B cells found CD86 expression to be similar or upregulated in CHBp compared to healthy individuals [118,119].

### 3.2. PD-1, PD-L1, and PD-L2

Programmed cell death protein 1, also known as PD-1 or CD279, is another immune checkpoint surface receptor, mainly expressed in T cells, NK cells, and B cells. PD-1 has two known ligands, PD-L1 (B7-H1 or CD274) and PD-L2 (B7-DC or CD273), which share a sequence homology of 37% [120]. PD-L1 is upregulated by antigen-presenting cells (APCs) and non-lymphoid tissues, like the liver, while PD-L2 is expressed by dendritic cells (DCs) and macrophages [121]. The primary role of PD-1 is to limit inflammatory responses against infections and prevent autoimmunity [56]. Unlike CTLA-4, the PD-1 pathway does not aim to dampen the initial activation of T cells but rather to control inflammatory responses in peripheral tissues by regulating effector T cells.

Upon T cell activation, PD-1 expression is induced, and T cells continue to express it in the tissues. Inflammatory signals in the tissues further enhance the expression of PD-L1 and PD-L2, which downregulate T cell activity and limit tissue damage in response to local infections [83]. When PD-1 engages with its ligands, it inhibits cytokine secretion, cell proliferation, and the cytotoxicity of effector immune cells [122].

Excessive induction of PD-1 in T cells during chronic exposure to viral antigens can lead to exhausted or anergic T cells [56]. Therefore, blocking the PD-1 pathway represents an interesting therapeutic approach with the potential to restore effector T cell responses against viral hepatitis and even HCC [121].

As expected, PD-1 was the most extensively studied immune checkpoint (Table S2—Supplementary Materials). Numerous studies have explored PD-1 expression not only on T cells but also on several other immune cell types.

Regarding CD4^+^ T cells, most of the research indicates a higher frequency of PD-1 expression in CHB patients compared to healthy individuals, although two studies did not report a significant difference. However, there is a relative lack of research focusing on the differences in PD-1 expression between CD4^+^ T cells from different compartments and subsets in CHB patients. Specifically, there is a need for more studies comparing PD-1 expression on CD4^+^ T cells from the liver versus the blood, as well as between total CD4^+^ T cells and those specific to the HBV.

CD8^+^ T cells were the most extensively studied immune cell subset, with several studies investigating PD-1 expression on total and HBV-specific cells. Similar to CD4^+^ T cells, CD8^+^ T cells from chronically infected patients tended to upregulate PD-1 compared to healthy subjects. Moreover, PD-1 expression was found to be stronger in intrahepatic CD8^+^ T cells than in circulating CD8^+^ T cells, and on HBV-specific CD8^+^ T cells compared to total CD8^+^ cells. Peripheral HBV-specific CD8^+^ T cells displayed an elevated frequency of PD-1 expression overall, although significantly lower than liver-infiltrating ones.

In studies on immune cells other than CD4^+^ and CD8^+^ T cells, the results have consistently shown greater PD-1 expression in CHB patients than in healthy controls, except for a few studies on Kupffer cells (KCs), regulatory T cells (Treg cells), and natural killer (NK) cells. In addition to elevated PD-1 expression on Th1 and Treg cells, Ji et al. also reported a significantly lower number of Th1 cells and DCs, and a higher number of Treg cells in the blood of CHB patients than in healthy individuals [123].

Immunohistochemical studies in CHB patients revealed that PD-1 is predominantly expressed on liver-infiltrating lymphocytes in the portal tract [124]. PD-L1 was detected in hepatocytes, liver sinusoidal endothelial cells, and lymphocytes, while PD-L2 was found on KCs and DCs [124].

Regarding PD-1 ligands, they were studied on various immune cell types, but their expression was generally low. However, both PD-L1 and PD-L2 were found to be overexpressed on liver-infiltrating lymphocytes (LILs) from CHB patients compared to healthy individuals. PD-L1 expression was also higher in peripheral blood mononuclear cells (PBMCs), monocytes, and DCs from infected subjects than in healthy individuals.

While CTLA-4 and PD-1 have been more extensively characterized, researchers have also reported results regarding other immune inhibitory receptors in CHB patients. These receptors include LAG-3, 2B4, KLRG-1, BTLA, CD160, TIGIT, Tim-3, and LAIR-1. While the number of studies on these receptors in CHB patients might be fewer compared to CTLA-4 and PD-1, their potential influence on immune response suppression cannot be dismissed.

### 3.3. LAG-3

Lymphocyte activation gene 3 (LAG-3, also known as CD223) is indeed another immune checkpoint that deserves consideration in the context of diseases characterized by T cell exhaustion [121]. It was first identified by Triebel et al. in 1990 [125], and is expressed on activated T cells, NK cells, B cells, and pDCs. LAG-3 interacts with antigen-presenting cells (APCs) through its major ligand, MHC Class II [126], and plays a central role in controlling T cell function and expansion. Blocking LAG-3 with specific monoclonal antibodies has been shown to enhance T cell activity [127]. 

Most studies on the expression of LAG-3 in CHB patients have focused on T cells (Table S3—Supplementary Materials). Kennedy et al. included CD4^+^ T cells in their experiments and found that the expression of LAG-3 on this T cell subset was low and similar between CHB patients and healthy adults [23]. On the other hand, conflicting results were reported for CD8^+^ T cells, with Ye et al. describing a reasonable and significantly higher LAG-3 expression in CHB patients than healthy adults, while Kennedy et al. found low and similar expression on both groups [23,128]. LAG-3 was upregulated on the surface of intrahepatic CD8^+^ T cells, whether considering total or HBV-specific cells [96,98]. The percentage of LAG-3^+^ circulating HBV-specific CD8^+^ T cells showed considerable discrepancy among studies [95,96]. Lastly, Fisicaro et al. found that around a quarter of the intrahepatic Treg cell population expressed LAG-3 on their surface [96]. In summary, the existing studies on LAG-3 expression in CHB patients are limited, and the results are somewhat inconsistent. More research is needed to draw definitive conclusions about LAG-3’s role in CHB and its potential as a therapeutic target for modulating immune responses and improving antiviral immunity in these patients.

### 3.4. 2B4 and CD48

Since its discovery in 1993, the 2B4 receptor (also known as CD244) has been described as an inhibitory surface molecule present in exhausted CD8^+^ T cells during chronic lymphocytic choriomeningitis virus (LCMV) infections [129,130]. CD244 belongs to the CD2 subset of the immunoglobulin superfamily and is expressed by innate immune cells such as NK cells, as well as CD8^+^ T cells [131]. 2B4 engages with CD48, which is typically expressed by hemopoietic cells [131]. The interaction between 2B4 and CD48 can lead to the development of T cells with impaired functions, as CD48 has a stronger affinity to 2B4 than CD2, a molecule needed for T cell activation, 

However, recent research has revealed that CD244 is a more multifunctional receptor than previously understood. While earlier reports suggested that 2B4 had a stimulatory role in both mice and humans, recent studies indicate that 2B4 may predominantly function as an inhibitory receptor [132]. The interaction between 2B4 and CD48 can provide both stimulatory and inhibitory effects, depending on the density of expression at the cell surface. Low to moderate expression of 2B4 may confer co-stimulatory features, while elevated expression can lead to inhibitory qualities. Furthermore, the regulatory role of 2B4 is influenced by the co-expression of other inhibitory receptors [133].

Most research on 2B4 expression is focused on CD8^+^ T cells and NK cells, as these cell subsets predominantly express this receptor (Table S4—Supplementary Materials). Overall, 2B4 expression tends to be consistently higher in intrahepatic CD8^+^ T cells compared to circulating ones, indicating a potentially more severe state of exhaustion in liver infiltrating CD8^+^ T cells compared to those in the peripheral blood. However, the expression of 2B4 on CD8^+^ T cells did not show significant differences between CHB patients and healthy adults, nor between total and HBV-specific CD8^+^ T cells from infected individuals.

Regarding NK cells, the reported results were more variable and inconclusive, making it challenging to draw definitive conclusions about 2B4 expression in CHB patients and healthy controls. Nevertheless, in some cases, a high proportion of both CD8^+^ T cells and NK cells expressed 2B4 on their surface, suggesting potential immune exhaustion. 

In the case of myeloid DCs, the expression of both 2B4 and its ligand CD48 was found to be similar between patients with persistent HBV infection and healthy controls. However, a study by Tjwa et al. found CD48 to be overexpressed in CHB patients relative to healthy adults [108].

Some researchers have also pointed out that the overexpression of 2B4 appears to be related to PD-1 upregulation, suggesting that modulating 2B4 alone may have less meaningful effects on T cell restoration compared to targeting other co-inhibitory receptors [121]. 

### 3.5. KLRG-1

Killer cell lectin-like receptor subfamily G member 1 (KLRG-1) is expressed by effector or memory T cells and differentiated NK cells [134]. The receptor binds to the ubiquitously expressed cadherins, cadherin-1 (CDH1, E-cadherin, or CD324) and cadherin-2 (CDH2, N-cadherin, or CD325), leading to inhibitory signaling [135]. As a result, KLRG-1-expressing cells demonstrate reduced proliferative capability and cytotoxic activity [135]. Consequently, the interaction between KLRG-1 and its ligands limits the antiviral activity of NK cells and T cells, contributing to immune exhaustion and viral persistence in chronic infections [134].

Research on KLRG-1 expression in chronically HBV-infected patients has mainly focused on T cells and NK cells (Table S5—Supplementary Materials). Both CD8^+^ T cells and NK cells displayed a moderate expression of KLRG-1, consistently higher in CHB patients compared to HAs. Interestingly, circulating HBV-specific CD8^+^ T cells showed a higher proportion of KLRG-1^+^ cells than the ones isolated from the liver of CHB patients. However, in NK cells, no significant difference was found in KLRG-1 expression between cells from peripheral blood and those from the liver. These findings highlight the role of KLRG-1 as an inhibitory receptor that contributes to immune exhaustion in chronic HBV infection. The upregulation of KLRG-1 on T cells and NK cells in CHB patients, compared to HAs, may be a signal of their compromised antiviral activity, contributing to the persistence of the virus. 

### 3.6. TIGIT

The T cell immunoglobulin and immunoreceptor tyrosine-based inhibitory motif domain, or TIGIT, is a co-inhibitory receptor expressed by NK cells and T cells, including memory T cells and Treg cells [136]. TIGIT interacts with its ligands, CD155 (also called PVR) and CD112, which are present on the surface of APCs [137]. Upon TIGIT–CD155 interaction, negative signaling suppresses T cell activity by increasing IL-10 secretion and reducing the release of proinflammatory cytokines [136]. TIGIT has been identified as a marker for exhausted T cells in various diseases, such as HIV, HCV, LCMV, and cancer [137].

While the contribution of TIGIT to HBV-related immune tolerance has not been extensively explored, recent studies have begun to shed light on this topic (Table S6—Supplementary Materials). Zong et al. observed a significantly higher percentage of TIGIT^+^CD8^+^ T cells in the blood of CHB patients compared to healthy adults [137]. Additionally, Schuch et al. reported broad expression of TIGIT on the surface of peripheral HBV-specific CD8^+^ T cells [138]. Further investigations are needed to fully elucidate the role of TIGIT in HBV-related immune dysfunction and its potential as a therapeutic target for immune restoration in chronic HBV infection.

### 3.7. BTLA and CD160

The receptors B and T lymphocyte attenuator (BTLA) and CD160 are part of a complex receptor–ligand network that involves the herpesvirus entry mediator (HVEM). BTLA and CD160 bind to HVEM, which also interacts with LIGHT and lymphotoxin-a (LTα) [139]. This complex network of receptors and ligands plays an essential role in regulating immune responses and maintaining immune homeostasis in various tissues and under different conditions. HVEM is often described as a “molecular switch” due to its ability to generate both co-stimulatory and co-inhibitory signals, depending on the ligands it interacts with. When HVEM binds to LIGHT and LTα, it induces co-stimulatory signals, promoting immune activation. On the other hand, when HVEM interacts with BTLA and CD160, it triggers co-inhibitory signals, dampening immune responses and contributing to immune tolerance [140].

Various lymphoid cells express CD160, BTLA, HVEM, LTα, and LIGHT at different stages of their lifetime, whether in an activated or resting state [140]. Furthermore, HVEM and its ligands can be found on both T cells and antigen-presenting cells (APCs) during the immune synapse [139]. BTLA, also known as CD272, exhibits high expression on activated T cells and resting B cells, with lower expression on NK cells, naive T cells, and some DCs and macrophages, suggesting that BTLA can transmit inhibitory signals to various immune cells [139]. It has a higher expression in Th1 than Th2 cells and a modest expression on Treg cells. Moreover, the high expression of BTLA on anergic T cells may indicate its crucial role in maintaining immune tolerance during chronic antigen exposure [141]. CD160 is predominantly expressed on γδ-T cells, NK cells, NKT cells, CD4^+^ and CD8^+^ T cells, and intestinal intraepithelial T lymphocytes (IELs), but it is not present on myeloid cells and B cells. Thus, CD160 and BTLA expressions only marginally overlap, suggesting that their suppressive functions may be distinct as well [140]. HVEM is highly expressed by resting T cells and immature or memory B cells, but it is downregulated on activated T and B cells [142]. HVEM is broadly expressed on DCs, monocytes, Treg cells, NK cells, and neutrophils, with the highest expression found in the kidney, lung, and liver [139]. LIGHT is expressed by activated T cells, granulocytes, and monocytes, as well as immature DCs, with its expression decreasing during DC maturation [143]. LTα, a soluble protein secreted by lymphocytes, can weakly engage with HVEM and shares TNFR1 and TNFR2 receptors with TNF-α [140]. 

Although HVEM has both co-stimulatory and co-inhibitory functions, its essential and non-redundant role is predominantly inhibitory, as demonstrated by the overactivation of lymphocytes in HVEM-deficient mice [139,144]. BTLA and CD160 are unique among immunoglobulin (Ig) domain-containing inhibitory receptors because they have the ability to bind to HVEM, a member of the TNFR superfamily. This highlights the flexibility of receptors, showing that they can interact with ligands belonging to different families [145]. The upregulation of BTLA is induced upon T-cell activation through TCR ligation, playing a crucial role in balancing activating and suppressing signals during immune responses [139]. BTLA and CD160 delivers a broad immune inhibition on T cells, suppressing proliferation, cytokines and cytokines receptors, and the expression of nutrient transport genes. Interestingly, the co-stimulatory and co-inhibitory ligands bind to different domains of the HVEM molecule [145]. BTLA and CD160 exert broad immune inhibition on T cells, suppressing proliferation, cytokine production, and cytokine receptor expression, as well as the expression of nutrient transport genes. 

The interactions between HVEM and its ligands form a complex and crucial receptor–ligand network that plays a key role in the regulation of immune tolerance and immune responses. However, among all the components involved in HVEM-dependent signaling, only BTLA and CD160 expressions have been studied in the context of the CHB population (Table S7—Supplementary Materials).

The existing literature on BTLA expression in CHB patients has yielded converging results regarding its similar expression on peripheral CD4^+^ T cells from both CHB patients and healthy adults. Also, Cai et al. found no significant difference in BTLA expression between the intrahepatic and circulating CD4^+^ T cells from HBV infected subjects [146]. 

Studies concerning BTLA expression in CD8^+^ T cells reported distinct results, since Cai et al. and Song et al. found similar expression levels between infected and healthy individuals, while Tang et al. found a higher proportion of BTLA^+^CD8^+^ T cells in the blood of chronically infected HBV patients [94,146,147]. Despite these discrepancies, it appears that there is a difference in BTLA expression levels between the peripheral blood and liver tissue of CHB patients, with higher levels observed in total and HBV-specific CD8^+^ T cells in the liver [146,148]. 

Treg cells, on the other hand, showed modest and equivalent peripheral BTLA expression in both CHB patients and healthy controls [149].

With respect to CD160, research has primarily concentrated on its expression in HBV-specific CD8^+^ T cells. Notably, among these studies, only Bengsch et al. reported that CD160 was upregulated on cells isolated from the liver compared to those from peripheral blood [95]. However, more research is needed to fully understand the role and expression patterns of CD160 in CHB patients. Overall, the regulation and expression of BTLA and CD160 in the context of CHB appear to be complex and may vary between different immune cell subsets and tissue locations.

### 3.8. Tim-3

T cell immunoglobulin and mucin-domain containing-3 (Tim-3) and its ligand, galectin-9 (Gal-9), have been gaining increasing attention as potential targets for cancer and infectious disease immunotherapy [150]. Tim-3 is highly and constitutively expressed on various immune cell types, including CD4^+^ Th1 and CD8^+^ cells, as well as on DCs, monocytes, NK cells, and macrophages [150,151,152]. Gal-9 is widely distributed in various organs, including lymphoid tissues, the liver, and the small intestine [153]. Tim-3 was initially identified as a negative regulator in chronic HIV infection, where Tim-3^+^ T cells were increased in infected patients, exhibiting impaired functions and proliferation. Blocking the Tim-3 pathway with a neutralizing antibody partially restored the anti-viral immune response in this context [154]. The interaction between Tim-3 and Gal-9 leads to T cell inhibition and apoptosis of Th1 cells, suggesting that the Tim-3–Gal-9 pathway plays a role in controlling T cell proliferation and tolerance, as well as preventing prolonged inflammation responses in the tissues [155].

Apart from CTLA-4 and PD-1, Tim-3 is one of the most extensively studied immune checkpoints in the context of CHB. Beyond the extensive research on T cells, Tim-3 expression has also been studied in various other immune cell types. In the case of CHB patients, studies have shown some discrepancies in Tim-3 expression when compared to healthy individuals. CD4^+^ T cells have generally shown low percentages of Tim-3^+^ cells, while CD8^+^ T cells tend to have a higher frequency of Tim-3^+^ cells in CHB patients compared to healthy controls (Table S8—Supplementary Materials). However, studies focused on HBV-specific CD8^+^ T cells have regularly reported low expression of Tim-3. Among other immune cell populations, such as monocytes, Treg cells, NK cells, and PBMCs, chronically infected HBV patients have been found to have higher Tim-3 expression compared to healthy individuals.

The growing attention to Tim-3 may be attributed to its elevated expression in various immune cell subsets during chronic HBV infection, as well as the presence of Gal-9 in the liver. Given that Tim-3 expression impedes a successful antiviral immune response, it is considered a potential target for the treatment of HBV infection [150]. Further research into the Tim-3–Gal-9 pathway and its regulatory role in immune responses may lead to novel therapeutic strategies for combating chronic HBV infection [156].

### 3.9. LAIR-1

Leukocyte-associated Ig-like receptor-1 (LAIR-1), also known as CD305, is another inhibitory receptor that can be exploited by viruses and tumor cells to evade the immune response. It belongs to the Ig superfamily and is expressed on the majority of thymocytes and peripheral blood mononuclear cells (PBMCs) [157]. LAIR-1 has been shown to interact with two tyrosine-protein phosphatases: PTPN11 and PTPN6 [158]. PTPN6 is predominantly expressed in hematopoietic cells, while PTPN11 is ubiquitously expressed across various cell types [159]. Activation of LAIR-1 in vitro has been demonstrated to inhibit cellular functions of B cells, NK cells, DC precursors, and effector T cells [157].

As of now, only two studies have been conducted on LAIR-1 expression in CHB patients (Table S9—Supplementary Materials). The first study reported high levels of LAIR-1 expression on total circulating CD4^+^ and CD8^+^ T cells in CHB patients, although the magnitude of expression was similar between CHB patients and healthy adults (HA) [23]. On the other hand, Gu et al. reported that LAIR-1 expression frequency on CD4^+^ T cells and CD8^+^ T cells, although at high levels on both parts, was significantly lower in patients with CHB when compared with HAs [160]. Further studies are needed to determine if LAIR-1 could potentially serve as a target for the treatment of chronic HBV infection. 

## 4. Expression of Cell Surface Co-Stimulatory Receptors in CHB

Activation of naive T cells requires two vital signals. The first signal is delivered by the interaction between the TCR and major histocompatibility complex (MHC) molecules, which allows T cells to recognize specific antigens. The second signal, known as co-stimulation, is equally important for the proper activation of T cells. One of the most crucial co-stimulatory pathways is the interaction between CD28 on T cells and B7 molecules (CD80 and CD86) on APCs. This interaction is considered the primary mechanism for stimulating naive T cells.

However, beyond the initial T cell activation, several other co-stimulatory molecules have been identified that play a role in amplifying and diversifying the T cell response. These molecules contribute to the development of effective immune responses against various pathogens and antigens [161]. 

In cases of impaired immune responses, such as in CHB patients, there is an elevation in co-inhibitory receptors and a potential downregulation of co-stimulatory molecules. This can lead to a synergistic effect, contributing to the exhaustion state of immune cells in CHB patients. To gain a deeper understanding of the immune response in CHB, numerous studies have explored the expression and function of various co-stimulatory molecules, including CD127, CD40, ICOS, 4-1BB, and OX40, in these patients. These studies aim to elucidate the intricate interaction between co-stimulatory and co-inhibitory pathways and their influence on the immune response in CHB infection. Understanding these mechanisms could help researchers identify potential targets for immunotherapeutic interventions to restore immune function in CHB patients.

### 4.1. CD127

Interleukin-7 (IL-7) and self-peptide/MHC ligands play critical roles in the development and maintenance of T cells, as well as in restoring the homeostasis of mature T cells [162]. IL-7 also possesses co-stimulatory effects and antagonizes TGF-β signaling [163]. While NK cells, B cells, and T cells cannot produce IL-7, certain cell types, like DCs, hepatocytes, neurons, and stromal cells, can produce this cytokine.

The IL-7 receptor (IL-7R) is a heterodimer composed of two subunits: the common γ-chain (CD132) and a high-affinity α-chain (CD127 or IL-7Rα) [164]. CD127 is not only crucial for T cell survival and proliferation but also plays a critical role in V(D)J recombination, a genetic recombination mechanism that generates the vast diversity of antibodies and T cell receptors (TCRs) found on B and T cells, respectively. This process occurs during the maturation of developing lymphocytes and is a fundamental characteristic of adaptive immunity [165]. 

CD127 is highly expressed on recent thymic mature T cells and is maintained on naive T cells. Upon T cell activation, CD127 is downregulated, but it is re-expressed on memory T cells [163]. However, Treg cells exhibit reduced expression of CD127, possibly due to the suppressive interaction between FoxP3 and the CD127 promoter [166].

In animal studies, IL-7 therapy has been shown to enhance immune responses to vaccines and improve viral clearance in acute and chronic infections. Several clinical trials have been registered, including the administration of recombinant human IL-7 for the treatment of HIV-, HBV-, or HCV-infected patients [163].

In CHB patients, CD127 is expressed in the majority of total and virus-specific CD4^+^ T cells (Table S10—Supplementary Materials). Raziorrouh et al. also reported that HBV-specific-CD127^high^CD4^+^ T cells displayed lower PD-1 expression and a decreased viral load, in contrast to HBV-specific-CD127^low^CD4^+^ T cells, suggesting a potential role in controlling the infection [97].

For CD8^+^ T cells, Fisicaro et al. reported similar CD127 expression on both total and HBV-specific CD8^+^ T cells, regardless of whether they were isolated from intrahepatic tissue or peripheral blood [87]. Furthermore, liver-infiltrating total and HBV-specific cells expressed less CD127, and more PD-1 compared to circulating cells, indicating a more severe state of T cell exhaustion at the site of HBV replication [87]. This suggests that continuous exposure to elevated antigen levels leads to the downregulation of CD127 and upregulation of PD-1 in CHB patients [87].

Finally, CD127 was moderately expressed on CD8^+^ memory T cells and Treg cells, with no significant difference between CHB patients and healthy individuals in the latter subset. These findings highlight the importance of IL-7 and its receptor in the immune response against chronic HBV infection.

### 4.2. CD40 and CD40L

CD40, a member of the TNF receptor superfamily, plays a pivotal role in immune cell function and maturation through its interaction with CD40 ligand (CD40L) [167]. CD40 is expressed on various immune cells, including macrophages, monocytes, B cells, dendritic cells (DCs), and activated T cells, as well as on non-immune cells. CD40L, primarily expressed by activated DCs, platelets, and T cells, also appears on monocytes, NK cells, and B cells [168]. 

The CD40–CD40L interaction at the immune synapse triggers complex events within antigen-presenting cells (APCs). Stimulation via CD40 causes complex events, such as B cell clonal expansion, affinity maturation and long-lived plasma cells formation [168]. Additionally, CD40–CD40L interaction regulates humoral and cell-mediated immunity, inflammation, cytokine production, adhesion molecule upregulation, and cell death induction [167]. As an adhesion molecule, CD40–CD40L interaction aids in prolonged T cell and APC interactions [161]. 

Disruption of the CD40–CD40L interaction can result in cellular and humoral immunodeficiency. Blocking this connection holds promise in abrogating autoimmune diseases and preventing transplant rejection [168].

As expected, studies regarding CD40 and CD40L were focused on APCs (DCs and B cells) and T cells, respectively (Table S11—Supplementary Materials). CD40 expression on CHB patients appears similar to healthy controls on both mDCs and pDCs, except for the findings by Martinet et al. [117], which showed increased CD40 expression on pDCs in CHB patients. Interestingly, total DCs and MoDCs showed a higher percentage of CD40^+^ cells in healthy controls. Despite conflicting results, both studies on CD40 expression on B cells reported elevated levels of this receptor on the cell surface.

Regarding CD40L, limited studies have been conducted in the CHB population. Existing research, however, indicates similar expression levels of CD40L on CD4^+^ and total T cells when comparing CHB patients and healthy controls, including HBV-specific cells.

### 4.3. ICOS and ICOSL

Inducible T-cell co-stimulator (ICOS, CD278) is a crucial co-stimulatory receptor expressed on activated T cells, which signals upon binding to its ligand, ICOSL (CD275) [169]. While ICOS is initially low on the surface of naive T cells, it rapidly increases following T-cell receptor (TCR) engagement and CD28 co-stimulatory signaling [170]. ICOSL is expressed by professional antigen-presenting cells (APCs), such as B cells, dendritic cells (DCs), and macrophages, as well as certain non-hematopoietic cells, like endothelial cells [169]. The interaction between ICOS and ICOSL induces T and B cell proliferation, cytokine production, and B cell differentiation into plasma cells. Moreover, ICOS plays a critical role in regulating local tissue responses to inflammation and co-stimulating memory T cell activity [169]. Moreover, ICOS can modulate immune responses and promote a Th1, Th2, or Th17 immunity by increasing the production of specific effector cytokines. Although ICOS and CD28 share some signaling pathways, ICOS co-stimulation is more modest than that triggered by CD28 [170]. 

Studies investigating ICOS expression in chronic hepatitis B (CHB) patients are limited (Table S12—Supplementary Materials). Expression of ICOS on CD4^+^ T cells was found to be low to moderate, with no significant differences between CHB patients and healthy controls, as well as between HBV-specific and total CD4^+^ T cells. Conversely, Tang et al. reported a significantly higher percentage of ICOS^+^CD8^+^ T cells in CHB patients compared to healthy controls [94]. 

Regarding ICOSL expression, results showed no significant difference between B cells and plasmacytoid DCs (pDCs) from CHB patients and healthy controls. 

### 4.4. 4-1BB and 4-1BBL

4-1BB (CD137 or TNF receptor superfamily member 9) is a co-stimulatory molecule expressed on CD4^+^ and CD8^+^ T cells, as well as on dendritic cells (DCs) and monocytes. Its primary ligand, 4-1BBL (CD137L), is expressed on antigen-presenting cells (APCs), like B cells, macrophages, and DCs, following stimulation [161]. The 4-1BB–4-1BBL interaction facilitates engagement between APCs and T cells, as well as between two APCs.

The 4-1BB–4-1BBL co-stimulatory pathway plays a critical role in T cell stimulation, preferentially enhancing CD8^+^ T cell expansion and promoting cytokine synthesis and T cell survival through anti-apoptotic mechanisms [171]. On the APCs’ side of the immune synapse, this interaction activates cytokine production and induces APC proliferation [161]. 4-1BB stimulation also enhances IFN-γ production, proliferation, and cytolytic activity in natural killer (NK) cells. In DCs, 4-1BB–4-1BBL engagement triggers the upregulation of some surface molecules, like CD80 and CD86, and promotes cell survival and IL-6 and IL-12 secretion [172]. Moreover, 4-1BB ligation can restore effector functions in impaired T cells with decreased cytotoxic ability [171].

Studies investigating 4-1BB expression in chronic hepatitis B (CHB) patients have primarily focused on various T cell populations (Table S13—Supplementary Materials). In CD4^+^ T cells, 4-1BB expression was generally low; however, Jacobi et al. still reported that HBV-specific cells had a lower 4-1BB^+^ frequency than total CD4^+^ T cells [173]. Fisicaro et al. found 4-1BB expression was found to be similar in HBV-specific CD8^+^ T cells isolated from both the liver and blood of CHB patients. Additionally, intrahepatic regulatory T cells (Treg cells) exhibited low levels of 4-1BB [96]. Regarding APCs, some studies reported higher 4-1BBL expression in B cells from CHB patients compared to healthy controls, while others found no significant difference in plasmacytoid DCs [117,174].

### 4.5. OX40 and OX40L

OX40 (TNFRSF4 or CD134) is a co-stimulatory immune checkpoint receptor that primarily binds to its ligand OX40L (CD252). While OX40 is predominantly expressed on activated CD4^+^ T cells, it is also found at lower levels on NK cells, NKT cells, and neutrophils [175]. OX40L expression is upregulated on APCs and some non-immune cells, as well as on activated CD4^+^ and CD8^+^ T cells [176]. Upon TCR binding to MHC molecules on CD4^+^ T cells, OX40 expression is augmented and further enhanced if co-stimulatory engagement of CD28 with CD80 and CD86 occurs [176].

OX40 stimulation promotes proliferation, effector molecule expression, and cytokine secretion by T cells [175]. OX40 signaling also modulates the function of Treg cells, impairing their suppressive ability and inhibiting FoxP3 expression, while diminishing the rate of conversion of naive T cells into Treg cells and antagonizing TGF-β. 

In CHB patients, studies have shown that OX40 expression on total CD4^+^ T cells is lower than on virus-specific cells, with similar levels observed between patients with persistent HBV infection and healthy individuals (Table S14—Supplementary Materials). However, OX40L expression on pDCs was found to be significantly lower in CHB patients compared to healthy controls [117].

## 5. Immune Checkpoint Inhibitors in HBV-Related Hepatocellular Carcinoma

Hepatocellular carcinoma (HCC) represents the most prevalent form of primary liver cancer and can be broadly categorized into viral and non-viral types based on its etiology. Viral related HCC includes cases induced by the HBV and the hepatitis C virus (HCV), whereas non-viral related HCC is typically associated with factors such as smoking, and alcoholism [177]. Early-stage HBV-induced HCC (HBV-HCC) can be addressed through conventional therapies, including locoregional therapies, surgery, and liver transplantation, and systemic therapies. However, due to the asymptomatic nature and the challenges in early diagnosis, most HCC cases are detected at an advanced stage, where treatment options are limited to systemic therapies with poor prognosis.

Immune checkpoint inhibitors (ICIs) have emerged as a promising treatment for advanced HCC by enhancing the anti-tumor immune response. These inhibitors work by activating T lymphocytes, thus boosting their ability to target and kill cancer cells. Strategies for immune checkpoint therapy in HBV-HCC encompass both monotherapy and combination therapies involving ICIs and targeted drugs. Clinical trials are currently underway, and several therapies are already approved, such as nivolumab, pembrolizumab, and combination therapy with atezolizumab plus bevacizumab and durvalumab plus tremelimumab [178]. Despite these efforts, the overall response rate to immunotherapy remains low, benefiting only a minority of patients with advanced HBV-HCC [179]. Furthermore, ICIs can cause adverse effects on vital organs, influenced by factors such as dosage tolerance and individual patient variability [180].

Nonetheless, further research is needed to fully harness the potential benefits of ICIs in the treatment of HBV-HCC.

## 6. Discussion

The blockade of immune checkpoints has emerged as a promising therapeutic strategy to restore the immune system in cancer and chronic infections. By harnessing the full potential of the immune response, antagonists of co-inhibitory receptors and agonists of co-stimulatory signals can synergistically amplify antigen-specific T cell responses. However, this approach faces significant challenges, including the risk of inducing autoimmunity and triggering uncontrolled hepatitis flares.

In the context of chronic hepatitis B (CHB) infection, antiviral therapies aimed at controlling HBV replication have shown only partial benefits in T cell function restoration. The limited recovery is attributed to persistently elevated antigen levels even after HBV DNA suppression. As conventional strategies may fall short in achieving a robust functional restoration, there is a need to explore additional therapeutic agents that directly target and stimulate virus-specific T cells for achieving complete T cell recovery [34].

Although various cells types are affected by a state of immune exhaustion, T cells have been the primary focus of immune modulatory therapeutics due to their unique ability to selectively recognize peptides, eliminate antigen-expressing cells (by CD8^+^ T cells), and coordinate diverse immune responses (by CD4^+^ T cells) [56].

Reversing T cell exhaustion by blocking the PD-1/PD-L1 pathway has demonstrated that exhausted T cells are not irreversibly terminal and can be restored to their normal function [47]. However, different subsets of dysfunctional CD8^+^ T cells with varying levels of expression of a specific immune checkpoint may respond differently to immune checkpoint inhibitors. For example, Blackburn et al. observed that CD8^+^ T cells with intermediate expression of PD-1 (PD-1^int^ cells) can be effectively reinvigorated upon PD-1 signaling blockade, unlike PD-1^high^ cells [181]. Hence, the potential improvement in T cell function following therapeutic intervention may be contingent on the PD-1^int^/PD-1^high^ T cell ratio [47].

The results of many authors indicate that the suppressive effects of the numerous inhibitory immune checkpoints on T cell exhaustion are non-redundant [34]. This suggests that dual blockade of specific immune checkpoint receptors or ligands could have synergistic effects in reconstituting T cells and enhancing host immunity. 

To date, antibodies targeting immune checkpoint pathways have been approved for the treatment of various diseases, including several types of cancer, such as lung cancer, melanoma, breast cancer, and hepatocellular carcinoma [92]. Clinical trials are also evaluating the potential of humanized monoclonal antibodies against PD-1 (nivolumab and pembrolizumab) and against CTLA-4 (tremelimumab and ipilimumab) for the treatment of HCC patients [121]. Notably, the PD-1 blockers, nivolumab and pembrolizumab, have already received approval from the Food and Drug Administration (FDA) and are currently being used as medication for HCC [92]. 

In 2019, a phase 2 clinical trial with the NCT number NCT04133259 was initiated to investigate the use of an immune checkpoint in CHB patients [181]. In this study, 44 subjects with CHB will be enrolled, and each participant will receive up to 3 doses of a recombinant anti-PD-1 humanized monoclonal antibody called HLX10, in combination with nucleos(t)ide analogue therapy [181]. The primary goals of the study are to evaluate the efficacy and safety of HLX10 by measuring the decline or seroclearance of HBsAg and the occurrence of hepatitis B flares in patients [182].

In conclusion, the increasing understanding of immune responses during chronic hepatitis B (CHB) infection is paving the way for novel approaches to restore abnormal innate immunity and recover impaired adaptive immunity. Enhanced insights into HBV pathogenesis provide scientists the opportunity to explore new strategies for modulating the immune system, with the ultimate goal of achieving lasting control over HBV infections. Immune checkpoint inhibitors (ICIs) hold significant promise in the treatment of CHB by reversing immune exhaustion and restoring effective antiviral immunity. Targeting key checkpoints, such as PD-1, CTLA-4, and Tim-3, can enhance T cell responses, making these inhibitors valuable components of combination therapies. The integration of ICIs with existing antiviral treatments and therapeutic vaccines represents a forward-looking strategy to achieve a functional cure for HBV. This approach addresses the complex challenges of chronic infection and paves the way for innovative treatment paradigms. Personalized medicine, through case-by-case screening of the most relevant immune checkpoints in each patient, can enhance the selection of the most appropriate ICI, optimizing treatment outcomes. This presents an exciting opportunity for researchers to design innovative and rational treatment strategies that combine antiviral agents with immunomodulatory molecules and other adjuvants. Such strategies have the potential to revolutionize the management of CHB, offering new hope for patients and advancing the field of HBV therapeutics.

## Figures and Tables

**Table 1:** CHB disease course and clinical characterization.

	Serum Ag	Antibodies	HBV DNA	ALT	Liver Histology
Phase 1: HBeAg-positive chronic HBV infection	HBeAg present	-	High	Normal	Near normal
Phase 2: HBeAg-positive chronic hepatitis B	HBeAg present	-	High	High fluctuating	Moderate/severe necroinflammation and fibrosis progression
Phase 3: HBeAg-negative chronic HBV infection	-	anti-HBe	Undetectable or low (<2000 IU/mL)	Normal	Minimal necroinflammation and low fibrosis
Phase 4: HBeAg-negative chronic hepatitis B	No HBeAg	anti-HBe	>2000 IU/ml	High fluctuating	Necroinflammation and fibrosis
Phase 5: HBsAg-negative phase	No HBsAg	anti-HBc	Usually undetectable	Normal	

## Supplementary Materials

The following supporting information can be downloaded at: https://www.mdpi.com/article/10.3390/ph17070964/s1, Table S1: Expression of CTLA-4, CD28, CD80 and CD86 from immune cells isolated from CHBp. Table S2: Expression of PD-1, PD-L1 and PD-L2 from immune cells isolated from CHBp. Table S3: Expression of LAG-3 from immune cells isolated from CHBp. Table S4: Expression of 2B4 and CD48 from immune cells isolated from CHBp. Table S5: Expression of KLRG-1 from immune cells isolated from CHBp. Table S6: Expression of TIGIT from immune cells isolated from CHBp. Table S7: Expression of BTLA and CD160 from immune cells isolated from CHBp. Table S8: Expression of Tim-3 from immune cells isolated from CHBp. Table S9: Expression of LAIR-1 from immune cells isolated from CHBp. Table S10: Expression of CD127 from immune cells isolated from CHBp. Table S11: Expression of CD40 and CD40L from immune cells isolated from CHBp. Table S12: Expression of ICOS and ICOSL from immune cells isolated from CHBp. Table S13: Expression of 4-1BB and 4-1BBL from immune cells isolated from CHBp. Table S14: Expression of OX40 and OX40L from immune cells isolated from CHBp.
