# Supplementary material for: Insights into Immune Exhaustion in Chronic Hepatitis B: A Review of Checkpoint Receptor Expression
Source: Pharmaceuticals (Basel). 2024 Jul 21;17(7):964. doi: 10.3390/ph17070964 (PMC11279883; doi:10.3390/ph17070964)
Supplement: Supplementary file 1 [file pharmaceuticals-17-00964-s001.zip › pharmaceuticals-3097347-supplementary.pdf]

Supplementary Material

Table S1: Expression of CTLA-4, CD28, CD80 and CD86 from immune cells isolated from CHBp.

	Cell subset	Comparison	Expression	Observation	Ref.
CTLA-4	CD4+ T cells	CHBp vs. HA	3.2 vs. 0.5 % [1]	Higher in CHBp than HA.	[1]
	CD4+ T cells	CHBp vs. HA	9.2 vs. 7.2 %	Higher in CHBp than HA.	[2]
	CD4+ T cells	CHBp vs. HA	120 vs. 230 x 10^6 CTLA-4+ cells/L [1]	Lower in CHBp than HA.	[3]
	CD4+ T cells	CHBp vs. HA	38 vs. 47 % [1]	Lower in CHBp than HA.	[4]
	CD4+ T cells	CHBp vs. HA	1 vs. 1 % [1]	No significant difference.	[5]
	CD4+ T cells	CHBp vs. HA	1.5 vs. 0.8 % [1]	Similar expression in CHBp and HA	[6]
	CD4+ T cells	CHBp only	3 % [1]	-	[7]
	HBV-specific CD4+ T cells	CHBp only	19.6 %	-	[8]
	CD8+ T cells	CHBp vs. HA	10 vs. 2 % [1]	Higher in CHBp than HA.	[1]
	CD8+ T cells	CHBp vs. HA	7 vs. 8 % [1]	No significant difference.	[4]
	CD8+ T cells	CHBp vs. HA	1.9 vs. 1.9 % [1]	No significant difference.	[5]
	CD8+ T cells	CHBp vs. HA	6 vs. 5.5 %	No significant difference.	[2]
	CD8+ T cells	CHBp vs. HA	0.2 vs. 0.2 %	No significant difference.	[6]
	CD8+ T cells	IH vs. PB	-	Higher in IH than PB.	[9]
	CD8+ T cells	HBV-specific vs. total	800 vs. 100 MFI [1]	Higher in HBV-specific than total.	[10]
	HBV-specific CD8+ T cells	IH vs. PB	17 vs. 22 % [1]	No significant difference.	[11]
	CD8+ T cells	CHBp only	4 % [1]	-	[7]
	HBV-specific CD8+ T cells	CHBp only	21.4 %	-	[12]
	MDSCs	CHBp vs. HA	5 vs. 2 % [1]	Higher in CHBp than HA.	[13]
	T reg cells	CHBp vs. HA	4 vs. 2 % [1]	Higher in CHBp than HA.	[14]
	T reg cells	CHBp vs. HA	56 vs. 55 %	No significant difference.	[2]
	T reg cells	IH vs. PB	70 vs. 30 % [1]	Higher in IH than PB.	[15]
	T reg cells	CHBp only	32.71 %	-	[16]
	IH T reg cells	CHBp only	75 % [1]	-	[11]
CD28	CD4+ T cells	CHBp vs HA	350 vs. 450 x 10^6 CD28+ cells/L [1]	Higher in CHBp than HA.	[3]
	CD4+ T cells	CHBp vs. HA	96 vs. 94.7 %	No significant difference.	[17]

	CD4+ T cells	CHBp vs. HA	97 vs. 97 % [1]	No significant difference.	[6]
	CD8+ T cells	CHBp vs. HA	78 vs. 85 % [1]	Lower in CHBp than HA.	[18]
	CD8+ T cells	CHBp vs. HA	51.1 vs. 51.3 %	No significant difference.	[17]
	CD8+ T cells	CHBp vs. HA	53 vs. 60 % [1]	No significant difference.	[6]
	CD8+ T cells	CHBp vs. HA	8.3 vs. 7.8 %	No significant difference.	[19]
	CD8+ T cells	CHBp only	5 % [1]	-	[20]
	iNKT cells	CHBp vs. HA	46.2 vs. 90.1 %	Lower in CHBp than HA.	[21]
CD80	DCs	CHBp vs. HA	25.75 vs. 38.52 %	Lower in CHBp than HA.	[22]
	DCs	CHBp vs. HA	44 vs. 88 %	Lower in CHBp than HA.	[23]
	DCs	CHBp vs. HA	36.86 vs. 59.87 %	Lower in CHBp than HA.	[24]
	DCs	CHBp vs. HA	37.62 vs. 11.98 %	Lower in CHBp than HA.	[25]
	DCs	CHBp only	33 %	-	[26]
	mDCs	CHBp vs. HA	600 vs. 200 MFI [1]	Higher in CHBp than HA.	[27]
	mDCs	CHBp vs. HA	47.9 vs. 72.5 %	Lower in CHBp than HA.	[28]
	mDCs	CHBp vs. HA	N.A.	No significant difference.	[29]
	mDCs	CHBp vs. HA	5 vs. 5 MFI [1]	No significant difference.	[30]
	Fresh mDCs	CHBp vs. HA	2.6 vs. 1.6 %	No significant difference.	[31]
	Matured mDCs	CHBp vs. HA	74 vs. 84 %	Lower in CHBp than HA.	[31]
	pDCs	CHBp vs. HA	5 vs. 5 MFI [1]	No significant difference.	[30]
	Fresh pDCs	CHBp vs. HA	1.8 vs. 1.6 %	No significant difference.	[31]
	Matured pDCs	CHBp vs. HA	54 vs. 51 %	No significant difference.	[31]
	Mature MoDCs	CHBp vs. HA	N.A.	Lower in CHBp than HA.	[29]
	MoDCs	CHBp only	47.9 %	-	[32]
	KCs	CHBp vs. HA	20 vs. 12.1 %	No significant difference.	[33]
	B cells	CHBp vs. HA	20 vs. 23 % [1]	Similar expression in CHBp and HA.	[34]
CD86	DCs	CHBp vs. HA	44 vs. 84 %	Lower in CHBp than HA.	[23]
	DCs	CHBp vs. HA	96.15 vs. 90.03 %	Lower in CHBp than HA.	[25]
	DCs	CHBp vs. HA	61.54 vs. 80.50 %	Lower in CHBp than HA.	[35]
	DCs	CHBp vs. HA	89.89 vs. 91.17 %	No significant difference.	[24]

	DCs	CHBp vs. HA	81.3 vs. 80.6 %	No significant difference.	[36]
	mDCs	CHBp vs. HA	2600 vs. 300 MFI [1]	Higher in CHBp than HA.	[27]
	mDCs	CHBp vs. HA	69.9 vs. 91.2 %	Lower in CHBp than HA.	[28]
	mDCs	CHBp vs. HA	82.6 vs. 75.4 %	No significant difference.	[37]
	mDCs	CHBp vs. HA	60 vs. 55 MFI [1]	No significant difference.	[30]
	mDCs	CHBp vs. HA	N.A.	No significant difference.	[29]
	Fresh mDCs	CHBp vs. HA	26 vs. 27 %	No significant difference.	[31]
	Matured mDCs	CHBp vs. HA	81 vs. 92 %	Lower in CHBp than HA.	[31]
	pDCs	CHBp vs. HA	35 vs. 15 % [1]	Higher in CHBp than HA.	[38]
	pDCs	CHBp vs. HA	28.8 vs. 17.0 %	No significant difference.	[37]
	pDCs	CHBp vs. HA	19 vs. 17 % [1]	No significant difference.	[39]
	pDCs	CHBp vs. HA	25 vs. 15 MFI [1]	No significant difference.	[30]
	Fresh pDCs	CHBp vs. HA	2.8 vs. 3.0	No significant difference.	[31]
	Matured pDCs	CHBp vs. HA	31 vs. 30 %	No significant difference.	[31]
	MoDCs	CHBp vs. HA	N.A.	Lower in CHBp than HA.	[29]
	KCs	CHBp vs. HA	22.8 vs. 14 %	Higher in CHBp than HA.	[33]
	B cells	CHBp vs. HA	18.41 vs. 8.22 %	Higher in CHBp than HA.	[40]
	B cells	CHBp vs. HA	9 vs. 11 % [1]	No significant difference.	[41]

CHBp: Chronic Hepatitis B patients; HA: Healthy Adults; IH: Intrahepatic; PB: Peripheral Blood; KC: Kupffer cells; iNKT cells: Invariant NKT cells; N.A.: Not available; [1]: Estimated values extrapolated from graphical analysis.

Table S2: Expression of PD-1, PD-L1 and PD-L2 from immune cells isolated from CHBp.

	Cell subset	Comparison	Expression	Observation	Ref.
PD-1	CD4+ T cells	CHBp vs. HA	33 vs. 23 % [1]	Higher in CHBp than HA.	[3]
	CD4+ T cells	CHBp vs. HA	22.9 vs. 15.8 %	Higher in CHBp than HA.	[2]
	CD4+ T cells	CHBp vs. HA	220 vs. 150 MFI [1]	Higher in CHBp than HA.	[42]
	CD4+ T cells	CHBp vs. HA	6.5 vs. 2.1 % [1]	Higher in CHBp than HA.	[43]
	CD4+ T cells	CHBp vs. HA	5.8 vs. 0.5 % [1]	Higher in CHBp than HA.	[5]
	CD4+ T cells	CHBp vs. HA	34.1 vs. 12.75 % [1]	Higher in CHBp than HA.	[44]
	CD4+ T cells	CHBp vs. HA	23 vs. 18 % [1]	Higher in CHBp than HA.	[45]
	CD4+ T cells	CHBp vs. HA	58.7 vs. 47.8 % [1]	Higher in CHBp than HA.	[46]
	CD4+ T cells	CHBp vs. HA	17 vs. 13 % [1]	Higher in CHBp than HA.	[47]
	CD4+ T cell	CHBp vs. HA	N.A.	Higher in CHBp than HA.	[48]
	CD4+ T cell	CHBp vs. HA	30 vs. 20 % [1]	Higher in CHBp than HA.	[49]
	CD4+ T cells	CHBp vs. HA	10000 vs. 4000 MFI [1]	Higher in CHBp than HA.<br>IFN-γ producing CD4+ T cells	[50]
	CD4+ T cells	CHBp vs. HA	20 vs. 29 % [1]	Higher in CHBp than HA.	[51]
	CD4+ T cells	CHBp vs. HA	40 vs. 36 % [1]	No significant difference.	[1]
	CD4+ T cells	CHBp vs. HA	22 vs. 23 % [1]	No significant difference.	[4]
	CD4+ T cells	CHBp vs. HA	5.5 vs. 4.9 % [1]	Similar expression in CHBp and HA.	[6]
	CD4+ T cells	IH vs. PB	57 vs. 8 % [1]	Higher in IH than PB.	[42]
	CD4+ T cells	HBV-specific vs. total	91 vs. 45 % [1]	Higher in HBV-specific than total.	[52]
	CD4+ T cells	HBV-specific vs. total	10000 vs. 10000 MFI [1]	No significant difference.<br>IFN-γ producing CD4+ T cells.	[50]
	CD4+ T cells	CHBp only	42.25 %	-	[53]
	CD4+ T cells	CHBp only	9.5 % [1]	-	[54]
	CD4+ T cells	CHBp only	6 % [1]	-	[7]
	HBV-specific CD4+ T cells	CHBp vs. HA	10000 vs. 4000 MFI [1]	Higher in CHBp than HA.<br>IFN-γ producing CD4+ T cells	[50]
	HBV-specific CD4+ T cells	CHBp only	77.9 %	-	[8]

	Th1 cells	CHBp vs. HA	523.1 vs. 437.5 MFI	Higher in CHBp than HA.	[55]
	CD8+ T cells	CHBp vs. HA	17 vs. 4 % [1]	Higher in CHBp than HA.	[3]
	CD8+ T cells	CHBp vs. HA	40 vs. 10 MFI [1]	Higher in CHBp than HA.	[56]
	CD8+ T cells	CHBp vs. HA	110 vs. 80 MFI [1]	Higher in CHBp than HA.	[42]
	CD8+ T cells	CHBp vs. HA	16.48 vs. 7.02 % [1]	Higher in CHBp than HA.	[44]
	CD8+ T cells	CHBp vs. HA	13.86 vs. 4.63 %	Higher in CHBp than HA.	[57]
	CD8+ T cells	CHBp vs. HA	21 vs. 17 % [1]	Higher in CHBp than HA.	[45]
	CD8+ T cells	CHBp vs. HA	6 vs. 1.7 % [1]	Higher in CHBp than HA.	[5]
	CD8+ T cells	CHBp vs. HA	38.5 vs. 25 %	Higher in CHBp than HA.	[1]
	CD8+ T cells	CHBp vs. HA	15 vs. 4 % [1]	Higher in CHBp than HA.	[58]
	CD8+ T cells	CHBp vs. HA	10 vs. 2 % [1]	Higher in CHBp than HA.	[59]
	CD8+ T cell	CHBp vs. HA	N.A.	Higher in CHBp than HA.	[48]
	CD8+ T cells	CHBp vs. HA	1.32 vs. 0.95 × 10^4/ml	Higher in CHBp than HA.	[60]
	CD8+ T cells	CHBp vs. HA	6.5 vs. 2.5 % [1]	Higher in CHBp than HA.	[43]
	CD4+ T cell	CHBp vs. HA	27 vs. 18 % [1]	Higher in CHBp than HA.	[49]
	CD8+ T cells	CHBp vs. HA	26000 vs. 7000 MFI [1]	Higher in CHBp than HA.<br>IFN-γ producing CD8+ T cells	[50]
	CD8+ T cells	CHBp vs. HA	6.5 vs. 6.4 % [1]	No significant difference.	[47]
	CD8+ T cells	CHBp vs. HA	18.8 vs. 20 %	No significant difference.	[2]
	CD8+ T cells	CHBp vs. HA	23 vs. 23 % [1]	No significant difference.	[61]
	CD8+ T cells	CHBp vs. HA	24 vs. 25 % [1]	No significant difference.	[4]
	CD8+ T cells	CHBp vs. HA	11 vs. 16 % [1]	No significant difference.	[62]
	CD8+ T cells	CHBp vs. HA	3.4 vs. 2.4 % [1]	Similar expression in CHBp and HA.	[6]
	CD8+ T cells	IH vs. PB	59 vs. 11 % [1]	Higher in IH than PB.	[42]
	CD8+ T cells	IH vs. PB	55 vs. 10 % [1]	Higher in IH than PB.	[63]
	CD8+ T cells	IH vs. PB	-	Higher in IH than PB.	[9]
	CD8+ T cells	HBV-specific vs. total	Genotype C: 37.4 vs. 4.74 % [1]<br>Genotype B: 26.3 vs. 2.55 % [1]	Higher in HBV-specific than total.	[64]
	CD8+ T cells	HBV-specific vs. total	110 vs. 40 MFI [1]	Higher in HBV-specific than total.	[56]

	CD8+ T cells	HBV-specific vs. total	28 vs. 15 % [1]	Higher in HBV-specific than total.	[65]
	CD8+ T cells	HBV-specific vs. total	55 vs. 4 % [1]	Higher in HBV-specific than total.	[66]
	CD8+ T cells	HBV-specific vs. total	71 vs. 31.2 %	Higher in HBV-specific than total.	[67]
	CD8+ T cells	HBV-specific vs. total	25 vs. 1 % [1]	Higher in HBV-specific than total.	[68]
	CD8+ T cells	HBV-specific vs. total	58 vs. 10 % [1]	Higher in HBV-specific than total.	[69]
	IH CD8+ T cells	HBV-specific vs. total	93 vs. 55 % [1]	Higher in HBV-specific than total.	[63]
	PB CD8+ T cells	HBV-specific vs. total	76.3 vs. 10 % [1]	Higher in HBV-specific than total.	[63]
	CD8+ T cells	HBV-specific vs. total	3.28 vs. 4.98 %	Similar expression.	[70]
	CD8+ T cells	HBV-specific vs. total	26000 vs. 31000 MFI [1]	Similar expression. IFN-γ producing CD8+ T cells	[50]
	CD8+ T cells	CHBp only	4.51 %	-	[71]
	CD8+ T cells	CHBp only	15 % [1]	-	[20]
	CD8+ T cells	CHBp only	12.5 % [1]	-	[54]
	CD8+ T cells	CHBp only	78 % [1]	-	[72]
	CD8+ T cells	CHBp only	6 % [1]	-	[7]
	CD8+ memory T cells	CHBp only	10 % [1]	-	[73]
	HBV-specific CD8+ T cells	CHBp vs. HA	31000 vs. 7000 MFI [1]	Higher in CHBp than HA. IFN-γ producing CD8+ T cells	[50]
	HBV-specific CD8+ T cells	IH vs. PB	93 vs. 76.3 %	Higher in IH than PB.	[63]
	HBV-specific CD8+ T cells	IH vs. PB	98 vs. 90 %	Higher in IH than PB.	[12]
	HBV-specific CD8+ T cells	IH vs. PB	85 vs. 55 % [1]	Higher in IH than PB.	[11]
	HBV-specific CD8+ T cells	CHBp only	31 %	-	[74]
	HBV-specific CD8+ T cells	CHBp only	45 % [1]	-	[61]
	HBV-specific CD8+ T cell	CHBp only	99 % [1]	core$_{18}$-specific CD8+ T cells	[75]
	HBV-specific CD8+ T cells	CHBp only	6 % [1]	-	[76]
	HBV-specific CD8+ T cells	CHBp only	78 – 96 %	-	[56]
	HBV-specific CD8+ T cells	CHBp only	64 – 100 % (24.4 – 66 MFI)	-	[77]
	HBV-specific CD8+ T cells	CHBp only	79.3 %	-	[12]
	T cells	CHBp only	8 % [1]	-	[78]
	T cells	CHBp only	80 % [1]	-	[79]

	PBMCs	CHBp vs. HA	67 vs. 54 MFI [1]	Higher in CHBp than HA.	[56]
	LIL	CHBp vs. HA	32.3 vs. 2.7 %	Higher in CHBp than HA.	[80]
	KCs	CHBp vs. HA	1.2 vs. 10.5 %	No significant difference.	[33]
	Monocytes	CHBp vs. HA	41 vs. 19 % [1]	Higher in CHBp than HA.	[81]
	B cells	CHBp vs. HA	41 vs. 10 % [1]	Higher in CHBp than HA.	[81]
	MDSCs	CHBp only	-	-	[82]
	T reg cells	CHBp vs. HA	32 vs. 21 % [1]	Higher in CHBp than HA.	[83]
	T reg cells	CHBp vs. HA	7164.1 vs. 1118.2 MFI	Higher in CHBp than HA.	[55]
	T reg cells	CHBp vs. HA	97.8 vs. 99.1 % [1]	No significant difference.	[46]
	T reg cells	IH vs. P	23 vs. 28 % [1]	No significant difference.	[15]
	T reg cells	CHBp only	25.6 / 12.5 %	eAg+ / eAg− patients	[72]
	T reg cells	CHBp only	5 % [1]	-	[84]
	IH T reg cells	CHBp only	78 % [1]	-	[11]
	NK cells	CHBp vs. HA	6300 vs. 2700 MFI [1]	Higher in CHBp than HA.	[85]
	NK cells	CHBp vs. HA	10 vs. 4 % [1]	Higher in CHBp than HA.	[59]
	NK cell	CHBp vs. HA	1900 vs. 2000 MFI	No significant difference.	[86]
	NK cells	CHBp vs. HA	4 vs. 7 % [1]	No significant difference.	[87]
	iNKT cells	CHBp vs. HA	84.5 vs. 35.5 %	Higher in CHBp than HA.	[21]
	iNKT cells	CHBp vs. HA	32 vs. 14 % [1]	Higher in CHBp than HA.	[88]
PD-L1	CD4+ T cells	CHBp only	0.93 %	-	[53]
	CD8+ T cells	HBV-specific vs. total	50 vs. 1 % [1]	Higher in HBV-specific than total.	[68]
	PBMCs	CHBp vs. HA	83 vs. 56 MFI [1]	Higher in CHBp than HA.	[56]
	LIL	CHBp vs. HA	30.7 vs. 5.3 %	Higher in CHBp than HA.	[80]
	Monocytes	CHBp vs. HA	5000 vs. 2300 MFI [1]	Higher in CHBp than HA.	[85]
	Monocytes	CHBp vs. HA	7 vs. 2 % [1]	Higher in CHBp than HA.	[89]
	DCs	CHBp vs. HA	3432.1 vs. 2010.3 MFI	Higher in CHBp than HA.	[55]
	MoDCs	CHBp only	10.7 %	-	[32]
	IH T reg cells	CHBp only	11 % [1]	-	[11]
	B cells	CHBp vs. HA	6 vs. 3 % [1]	No significant difference.	[90]
	MDSCs	CHBp vs. HA	10 vs. 5 % [1]	Higher in CHBp than HA.	[13]
PD-L2	CD8+ T cells	HBV-specific vs. total	58 vs. 1 % [1]	Higher in HBV-specific than total.	[68]

	PBMCs	CHBp vs. HA	-	Low and no significant difference in expression.	[56]
	LIL	CHBp vs. HA	15.7 vs. 2.1 %	Higher in CHBp than HA.	[80]

CHBp: Chronic Hepatitis B patients; HA: Healthy Adults; IH: Intrahepatic; PB: Peripheral Blood; iNKT cells: Invariant NKT cells; N.A.: Not available; LIL: Liver Infiltrating Lymphocytes; [1]: Estimated values extrapolated from graphical analysis.

Table S3: Expression of LAG-3 from immune cells isolated from CHBp.

	Cell subset	Comparison	Expression	Observation	Ref.
LAG-3	CD4+ T cells	CHBp vs. HA	32 vs. 39 % [1]	Higher in CHBp than HA.	[51]
	CD4+ T cells	CHBp vs. HA	2.1 vs. 1.8 % [1]	No significant difference.	[5]
	CD4+ T cells	CHBp vs. HA	3 vs. 2 % [1]	No significant difference.	[4]
	CD8+ T cells	CHBp vs. HA	41.15 vs. 25.96 %	Higher in CHBp than HA.	[91]
	CD8+ T cells	CHBp vs. HA	2.1 vs. 2 % [1]	No significant difference.	[5]
	CD8+ T cells	CHBp vs. HA	2 vs. 2 % [1]	No significant difference.	[4]
	CD8+ T cells	IH vs. PB	-	Higher in IH than PB.	[9]
	HBV-specific CD8+ T cells	IH vs. PB	50 vs. 32 % [1]	Higher in IH than PB.	[11]
	HBV-specific CD8+ T cells	CHBp only	0.9 %	-	[12]
	IH T reg cells	CHBp only	27 % [1]	-	[11]

CHBp: Chronic Hepatitis B patients; HA: Healthy Adults; IH: Intrahepatic; PB: Peripheral Blood; N.A.: Not available; [1]: Estimated values extrapolated from graphical analysis.

Table S4: Expression of 2B4 and CD48 from immune cells isolated from CHBp.

	Cell subset	Comparison	Expression	Observation	Ref.
2B4	CD4+ T cells	CHBp vs. HA	1200 vs. 1250 MFI [1]	No significant difference. IFN-γ producing CD4+ T cells	[50]
	CD4+ T cells	CHBp vs. HA	120 vs. 17 % [1]	No significant difference.	[4]
	CD4+ T cells	CHBp vs. HA	13 vs. 13 % [1]	No significant difference.	[51]
	CD4+ T cells	HBV-specific vs. total	1450 vs. 1200 MFI [1]	Similar expression. IFN-γ producing CD4+ T cells	[50]
	HBV-specific CD4+ T cells	CHBp vs. HA	1450 vs. 1000 MFI [1]	Higher in CHBp than HA. IFN-γ producing CD4+ T cells	[50]
	CD4+ T cells	CHBp only	12 % [1]	-	[92]
	HBV-specific CD4+ T cells	CHBp only	5.5 %	-	[8]
	CD8+ T cells	CHBp vs. HA	20 vs. 70 % [1]	Lower in CHBp than HA.	[4]
	CD8+ T cells	CHBp vs. HA	51 vs. 48 % [1]	No significant difference.	[93]
	CD8+ T cells	CHBp vs. HA	2400 vs. 1700 MFI [1]	No significant difference. IFN-γ producing CD8+ T cells	[50]
	CD8+ T cells	IH vs. PB	91.7 vs. 50 %	Higher in IH than PB	[93]
	CD8+ T cells	HBV-specific vs. total	2000 vs. 2400 MFI [1]	Similar expression. IFN-γ producing CD8+ T cells	[50]
	CD8+ T cell	HBV-specific vs. total	62 vs. 41 % [1]	Higher in HBV-specific than total.	[68]
	IH CD8+ T cells	HBV-specific vs. total	97 vs. 91.7 %	No significant difference.	[93]
	HBV-specific CD8+ T cells	CHBp vs. HA	2000 vs. 1500 MFI [1]	Higher in CHBp than HA. IFN-γ producing CD8+ T cells	[50]
	HBV-specific CD8+ T cells	IH vs. PB	97 vs. 78 %	Higher in IH than PB	[93]
	HBV-specific CD8+ T cells	IH vs. PB	90 vs. 50 % [1]	Higher in IH than PB	[12]
	HBV-specific CD8+ T cells	IH vs. PB	80 vs. 50 % [1]	No significant difference.	[11]
	CD8+ T cells	CHBp only	73 % [1]	-	[92]
	HBV-specific CD8+ T cells	CHBp only	53.9 %	-	[12]
	HBV-specific CD8+ T cell	CHBp only	97 % [1]	$core_{18}$-specific CD8+ T cells	[75]
	CD8+ memory T cells	CHBp only	5 % [1]	-	[73]
	NK cell	CHBp vs. HA	145 vs. 70 MFI [1]	Higher in CHBp than HA	[27]
	NK cell	CHBp vs. HA	0.59 vs. 1.80 x 10^4/ml	Lower in CHBp than HA	[60]
	NK cell	CHBp vs. HA	1.1 vs. 1.5 x 10^4/ml	Lower in CHBp than HA	[94]
	NK cell	CHBp vs. HA	98 vs. 83 % [1]	No significant difference.	[95]
	NK cell	CHBp vs. HA	93 vs. 97 % [1]	No significant difference.	[96]
	NK cell	CHBp only	90 % [1]	-	[92]

	mDCs	CHBp vs. HA	120 vs. 110 MFI [1]	No significant difference.	[27]
CD48	NK cell	CHBp vs. HA	150 vs. 120 MFI [1]	Higher in CHBp than HA	[27]
	mDCs	CHBp vs. HA	210 vs 270 MFI [1]	No significant difference.	

CHBp: Chronic Hepatitis B patients; HA: Healthy Adults; IH: Intrahepatic; PB: Peripheral Blood; N.A.: Not available; [1]: Estimated values extrapolated from graphical analysis.

Table S5: Expression of KLRG-1 from immune cells isolated from CHBp.

	Cell subset	Comparison	Expression	Observation	Ref.
KLRG-1	CD4+ T cell	CHBp vs. HA	N.A.	No significant difference.	[48]
	HBV-specific CD4+ T cells	CHBp only	6.4 %	-	[8]
	CD8+ T cell	CHBp vs. HA	N.A.	Higher in CHBp than HA.	[48]
	HBV-specific CD8+ T cell	IH vs. PB	35 vs. 55 % [1]	Lower in IH than PB.	[12]
	HBV-specific CD8+ T cell	CHBp only	61.7 %	-	[12]
	HBV-specific CD8+ T cell	CHBp only	52 % [1]	$core_{18}$-specific CD8+ T cells	[75]
	NK cells	CHBp vs. HA	13.4 vs. 2.3 %	Higher in CHBp than HA.	[97]
	NK cells	IH vs. PB	22 vs. 19 % [1]	No significant difference.	[97]
	IH NK cells	CHBp vs. HA	23.4 vs. 2.6 %	Higher in CHBp than HA.	[97]

CHBp: Chronic Hepatitis B patients; HA: Healthy Adults; IH: Intrahepatic; PB: Peripheral Blood; N.A.: Not available; [1]: Estimated values extrapolated from graphical analysis.

Table S6: Expression of TIGIT from immune cells isolated from CHBp.

	Cell subset	Comparison	Expression	Observation	Ref.
TIGIT	CD8+ T cell	CHBp vs. HA	30 vs. 24 % [1]	Higher in CHBp than HA.	[98]
	CD8+ T cell	HBV-specific vs. total	65 vs. 27 % [1]	Higher in HBV-specific than total.	[68]
	HBV-specific CD8+ T cell	CHBp only	96 % [1]	$core_{18}$-specific CD8+ T cells	[75]
	NK cell	CHBp vs. HA	68 vs. 62 % [1]	No significant difference.	[99]

CHBp: Chronic Hepatitis B patients; HA: Healthy Adults; [1]: Estimated values extrapolated from graphical analysis.

Table S7: Expression of BTLA and CD160 from immune cells isolated from CHBp.

	Cell subset	Comparison	Expression	Observation	Ref.
BTLA	CD4+ T cells	CHBp vs. HA	10 vs. 18 % [1]	Higher in CHBp than HA.	[100]
	CD4+ T cells	CHBp vs. HA	2.8 vs. 2.4 % [1]	No significant difference.	[46]
	CD4+ T cells	CHBp vs. HA	21 vs. 19 % [1]	No significant difference.	[6]
	CD4+ T cells	CHBp vs. HA	3200 vs. 2800 MFI [1]	No significant difference.	[42]
	CD4+ T cells	IH vs. PB	77 vs. 76 % [1]	No significant difference.	[42]
	CD8+ T cells	CHBp vs. HA	10 vs. 4.5 % [1]	Higher in CHBp than HA	[6]
	CD8+ T cells	CHBp vs. HA	40 vs. 7 % [1]	Higher in CHBp than HA	[100]
	CD8+ T cells	CHBp vs. HA	1900 vs. 1200 MFI [1]	No significant difference.	[42]
	CD8+ T cells	IH vs. PB	55 vs. 35 % [1]	Higher in IH than PB.	[42]
	CD8+ T cells	HBV-specific vs. total	30.6 vs. 72.5 %	Lower in HBV-specific than total. Values from one representative patient.	[101]
	HBV-specific CD8+ T cells	IH vs. PB	0.1 – 9 vs. 0.02 – 0.8 % [1]	Higher in IH than PB.	[101]
	HBV-specific CD8+ T cells	CHBp only	6.2 %	-	[12]
	T reg cells	CHBp vs. HA	9.8 vs. 14.0 % [1]	No significant difference.	[46]
CD160	CD4+ T cells	CHBp vs. HA	5 vs. 6 % [1]	No significant difference.	[51]
	HBV-specific CD8+ T cells	IH vs. PB	50 vs. 55 % [1]	No significant difference.	[11]
	HBV-specific CD8+ T cells	IH vs. PB	37 vs. 5 % [1]	Higher in IH than PB.	[12]
	HBV-specific CD8+ T cells	CHBp only	25.1 %	-	[12]

CHBp: Chronic Hepatitis B patients; HA: Healthy Adults; IH: Intrahepatic; PB: Peripheral Blood; [1]: Estimated values extrapolated from graphical analysis.

Table S8: Expression of Tim-3 from immune cells isolated from CHBp.

	Cell subset	Comparison	Expression	Observation	Ref.
Tim-3	CD4+ T cells	CHBp vs. HA	12 vs. 6 % [1]	Higher in CHBp than HA.	[102]
	CD4+ T cells	CHBp vs. HA	6.41 vs. 3.32 %	Higher in CHBp than HA.	[103]
	CD4+ T cells	CHBp vs. HA	2.9 vs. 1.5 % [1]	Higher in CHBp than HA.	[104]
	CD4+ T cells	CHBp vs. HA	3.93 vs. 1.48 %	Higher in CHBp than HA.	[105]
	CD4+ T cells	CHBp vs. HA	180 vs. 80 x 10^6/l	Higher in CHBp than HA.	[3]
	CD4+ T cells	CHBp vs. HA	2.3 vs. 1.9 % [1]	No significant difference.	[5]
	CD4+ T cells	CHBp vs. HA	28 vs. 27 % [1]	No significant difference.	[1]
	CD4+ T cells	CHBp vs. HA	76 vs. 80 % [1]	No significant difference.	[4]
	CD4+ T cells	CHBp vs. HA	600 vs. 600 MFI [1]	No significant difference. IFN-γ producing CD4+ T cells	[50]
	CD4+ T cells	IH vs. PB	10-33 vs. 5-19 % [1]	Higher in IH than PB.	[102]
	CD4+ T cells	HBV-specific vs. total	770 vs. 600 MFI [1]	Similar expression. IFN-γ producing CD4+ T cells	[50]
	HBV-specific CD4+ T cells	CHBp vs. HA	770 vs. 620 MFI [1]	No significant difference. IFN-γ producing CD4+ T cells	[50]
	HBV-specific CD4+ T cells	CHBp only	12.9 %	-	[8]
	Th17 cells	CHBp vs. HA	3.2 vs. 0.8 % [1]	Higher in CHBp than HA.	[106]
	CD8+ T cells	CHBp vs. HA	19 vs. 6 % [1]	Higher in CHBp than HA.	[104]
	CD8+ T cells	CHBp vs. HA	21.2 vs. 10.1 %	Higher in CHBp than HA.	[102]
	CD8+ T cells	CHBp vs. HA	4.72 vs. 2.28 %	Higher in CHBp than HA.	[103]
	CD8+ T cells	CHBp vs. HA	90 vs. 5 x 10^6/l	Higher in CHBp than HA.	[3]
	CD8+ T cells	CHBp vs. HA	21.34 vs. 6.33 %	Higher in CHBp than HA.	[105]
	CD8+ T cells	CHBp vs. HA	5 vs. 10 % [1]	Higher in CHBp than HA.	[99]
	CD8+ T cells	CHBp vs. HA	45 vs. 55 % [1]	Lower in CHBp than HA.	[1]
	CD8+ T cells	CHBp vs. HA	4.2 vs. 6 % [1]	No significant difference.	[5]
	CD8+ T cells	CHBp vs. HA	77 vs. 73 % [1]	No significant difference.	[4]
	CD8+ T cells	CHBp vs. HA	1000 vs. 1400 MFI [1]	No significant difference. IFN-γ producing CD4+ T cells	[50]

	CD8+ T cells	IH vs. PB	18-45 vs. 13-34 % [1]	Higher in IH than PB.	[102]
	CD8+ T cells	IH vs. PB	N.A.	Higher in IH than PB.	[9]
	CD8+ T cells	HBV-specific vs. total	31 vs. 21.2 % [1]	Higher in HBV-specific than total.	[102]
	CD8+ T cells	HBV-specific vs. total	990 vs. 1000 MFI [1]	Similar expression. IFN-γ producing CD4+ T cells	[50]
	CD8+ T cells	CHBp only	8.43 %	-	[71]
	HBV-specific CD8+ T cells	CHBp vs. HA	990 vs. 920 MFI [1]	No significant difference. IFN-γ producing CD4+ T cells	[50]
	HBV-specific CD8+ T cells	IH vs. PB	2 vs. 7 % [1]	Similar expression.	[12]
	HBV-specific CD8+ T cells	CHBp only	4 % [1]	-	[76]
	HBV-specific CD8+ T cells	CHBp only	7.5 %	-	[12]
	HBV-specific CD8+ T cells	CHBp only	0.3 %	-	[104]
	T cells	CHBp only	65 % [1]	-	[79]
	Monocytes	CHBp vs. HA	57.7 vs. 49 %	Higher in CHBp than HA.	[107]
	Monocytes	CHBp vs. HA	15 vs. 5 % [1]	Higher in CHBp than HA.	[106]
	T reg cells	CHBp vs. HA	9.6 vs. 4.1 %	Higher in CHBp than HA.	[108]
	T reg cells	IH vs. PB	61 vs. 6.8 %	Higher in IH than PB.	[108]
	NK cell	CHBp vs. HA	5000 vs. 4000	Higher in CHBp than HA.	[86]
	NK cells	CHBp vs. HA	68 vs. 59 %	Higher in CHBp than HA.	[109]
	NK cells	CHBp vs. HA	5 vs. 20 % [1]	Higher in CHBp than HA.	[87]
	NK cells	CHBp only	80 % [1]	-	[110]
	iNKT cells	CHBp vs. HA	89.4 vs. 23.3 %	Higher in CHBp than HA.	[21]
	NKT-like cells	CHBp vs. HA	40.6 vs. 30.7 %	Higher in CHBp than HA.	[107]
	NKT cells	CHBp only	80 % [1]	-	[110]
	PBMCs	CHBp vs. HA	18.53 vs. 15.47 %	Higher in CHBp than HA.	[109]
	PBMCs	CHBp vs. HA	27 vs. 18 % [1]	Higher in CHBp than HA.	[104]

CHBp: Chronic Hepatitis B patients; HA: Healthy Adults; IH: Intrahepatic; PB: Peripheral Blood; NKT-like cells: Natural Killer-like T cells; iNKT cells: Invariant NKT cells; PBMCs: Peripheral Blood Mononuclear Cells; [1]: Estimated values extrapolated from graphical analysis.

Table S9: Expression of LAIR-1 from immune cells isolated from CHBp.

	Cell subset	Comparison	Expression	Observation	Ref.
LAIR-1	CD4+ T cells	CHBp vs. HA	80 vs. 81 % [1]	No significant difference.	[5]
	CD4+ T cells	CHBp vs. HA	65.3 vs. 74.3 %	Lower in CHBp than HA.	[4]
	CD8+ T cells	CHBp vs. HA	80 vs. 85 % [1]	No significant difference.	[5]
	CD8+ T cells	CHBp vs. HA	82.4 vs. 92.1 %	Lower in CHBp than HA.	[4]

CHBp: Chronic Hepatitis B patients; HA: Healthy Adults; [1]: Estimated values extrapolated from graphical analysis.

Table S10: Expression of CD127 from immune cells isolated from CHBp.

	Cell subset	Comparison	Expression	Observation	Ref.
CD127	CD4+ T cells	HBV-specific vs. total	80 vs. 80 % [1]	No significant difference.	[52]
	HBV-specific CD4+ T cells	CHBp only	54.8 %	-	[8]
	CD8+ T cells	CHBp vs. HA	N.A.	Similar expression.	[111]
	CD8+ T cells	IH vs.PB	27 vs. 72 % [1]	Lower in IH than PB.	[63]
	CD8+ T cells	HBV-specific vs. total	71 vs. 72 % [1]	No significant difference.	[63]
	CD8+ T cell	HBV-specific vs. total	13 vs. 51 % [1]	Lower in HBV-specific than total.	[68]
	IH CD8+ T cells	HBV-specific vs. total	28.2 vs 27 % [1]	No significant difference.	[63]
	HBV-specific CD8+ T cells	IH vs.PB	28.2 vs. 71 %	Lower in IH than PB.	[63]
	HBV-specific CD8+ T cells	CHBp only	63 – 100 %	-	[77]
	HBV-specific CD8+ T cells	CHBp only	6 % [1]	-	[111]
	CD8+ memory T cells	CHBp only	54 % [1]	-	[73]
	CD8+ memory T cells	CHBp only	25 % [1]	-	[111]
	T reg cells	CHBp vs. HA	18 vs. 17 %	No significant difference.	[2]

CHBp: Chronic Hepatitis B patients; HA: Healthy Adults; IH: Intrahepatic; PB: Peripheral Blood; N.A.: Not available; [1]: Estimated values extrapolated from graphical analysis.

Table S11: Expression of CD40 and CD40L from immune cells isolated from CHBp.

	Cell subset	Comparison	Expression	Observation	Ref.
CD40	mDCs	CHBp vs. HA	81.6 vs. 89.3 %	No significant difference.	[28]
	mDCs	CHBp vs. HA	25 vs. 5 MFI [1]	No significant difference.	[30]
	mDCs	CHBp vs. HA	21.5 vs. 22 %	Similar expression.	[37]
	mDCs	CHBp vs. HA	N.A.	No significant difference.	[29]
	pDCs	CHBp vs. HA	90 vs. 80 % [1]	Higher in CHBp than HA.	[38]
	pDCs	CHBp vs. HA	25 vs. 15 MFI [1]	No significant difference.	[30]
	pDCs	CHBp vs. HA	15.3 vs. 6 %	Similar expression.	[37]
	MoDCs	CHBp vs. HA	23 vs. 44 %	Lower in CHBp than HA.	[112]
	MoDCs	CHBp only	81.6 %	-	[32]
	DCs	CHBp vs. HA	40.01 vs. 60.00 %	Lower in CHBp than HA.	[35]
	B cells	CHBp vs. HA	94 vs. 98 % [1]	Lower in CHBp than HA.	[113]
	B cells	CHBp vs. HA	93 vs. 90 % [1]	Similar expression.	[90]
	MDSCs	CHBp vs. HA	7 vs. 3 % [1]	Higher in CHBp than HA.	[13]
CD40L	CD4+ T cells	CHBp vs. HA	8 vs. 7 %	Similar expression.	[114]
	CD4+ T cells	HBV-specific vs. total	12 vs. 11 % [1]	No significant difference.	[52]
	T cells	CHBp vs. HA	96 vs. 100 MFI	Similar expression.	[115]

CHBp: Chronic Hepatitis B patients; HA: Healthy Adults; N.A.: Not available; [1]: Estimated values extrapolated from graphical analysis.

Table S12: Expression of ICOS and ICOSL from immune cells isolated from CHBp.

	Cell subset	Comparison	Expression	Observation	Ref.
ICOS	CD4+ T cells	CHBp vs. HA	5 vs. 5 % [1]	No significant difference.	[6]
	CD4+ T cells	HBV-specific vs. total	18 vs. 20 % [1]	No significant difference.	[52]
	CD8+ T cells	CHBp vs. HA	1 vs. 0.5 % [1]	Higher in CHBp than HA.	[6]
	CD8+ T cells	CHBp vs. HA	1 vs. 15 % [1]	Higher in CHBp than HA.	[68]
ICOSL	B cells	CHBp vs. HA	4.5 vs. 3.5 % [1]	No significant difference.	[90]
	pDCs	CHBp vs. HA	0.3 vs. 0.2 % [1]	No significant difference.	[38]

CHBp: Chronic Hepatitis B patients; HA: Healthy Adults; [1]: Estimated values extrapolated from graphical analysis.

Table S13: Expression of 4-1BB and 4-1BBL from immune cells isolated from CHBp.

	Cell subset	Comparison	Expression	Observation	Ref.
4-1BB	CD4+ T cells	HBV-specific vs. total	4 vs. 10 % [1]	Lower in HBV-specific than total.	[52]
	CD4+ T cells	CHBp only	1.7 % [1]	-	[116]
	CD8+ T cells	CHBp only	2.2 % [1]	-	[116]
	CD8+ T cells	HBV-specific vs. total	13 vs. 1 % [1]	Higher in HBV-specific than total.	[68]
	HBV-specific CD8+ T cells	IH vs. PB	50 vs. 45 % [1]	No significant difference.	[11]
	T cells	CHBp vs. HA	1.8 vs. 1.7 % [1]	No significant difference.	[116]
	IH T reg cells	CHBp only	5 % [1]	-	[11]
4-1BBL	B cells	CHBp vs. HA	6 vs. 3 % [1]	Higher in CHBp than HA.	[116]
	pDCs	CHBp vs. HA	0.4 vs. 0.6 % [1]	No significant difference.	[38]

CHBp: Chronic Hepatitis B patients; HA: Healthy Adults; IH: Intrahepatic; PB: Peripheral Blood; [1]: Estimated values extrapolated from graphical analysis.

Table S14: Expression of OX40 and OX40L from immune cells isolated from CHBp.

	Cell subset	Comparison	Expression	Observation	Ref.
OX40	CD4+ T cells	CHBp vs. HA	12 vs. 9 % [1]	Similar expression.	[117]
	CD4+ T cells	HBV-specific vs. total	82 vs. 30 % [1]	Higher in HBV-specific than total.	[52]
OX40L	pDCs	CHBp vs. HA	7 vs. 22 % [1]	Lower in CHBp than HA.	[38]

CHBp: Chronic Hepatitis B patients; HA: Healthy Adults; [1]: Estimated values extrapolated from graphical analysis.

References

1. Yong YK, Saeidi A, Tan HY, Rosmawati M, Enstrom PF, Batran RA, et al. Hyper-Expression of PD-1 Is Associated with the Levels of Exhausted and Dysfunctional Phenotypes of Circulating CD161(++)TCR iValpha7.2(+) Mucosal-Associated Invariant T Cells in Chronic Hepatitis B Virus Infection. Front Immunol. 2018;9:472.
2. Park JJ, Wong DK, Wahed AS, Lee WM, Feld JJ, Terrault N, et al. Hepatitis B Virus--Specific and Global T-Cell Dysfunction in Chronic Hepatitis B. Gastroenterology. 2016;150(3):684-95 e5.
3. Wang L, Zhao C, Peng Q, Shi J, Gu G. Expression levels of CD28, CTLA-4, PD-1 and Tim-3 as novel indicators of T-cell immune function in patients with chronic hepatitis B virus infection. Biomed Rep. 2014;2(2):270-4.
4. Gu Y, Bi Y, Wei H, Li J, Huang Z, Liao C, et al. Expression and clinical significance of inhibitory receptor Leukocyte-associated immunoglobulin-like receptor-1 on peripheral blood T cells of chronic hepatitis B patients: A cross-sectional study. Medicine. 2021;100(29):e26667.
5. Kennedy PTF, Sandalova E, Jo J, Gill U, Ushiro-Lumb I, Tan AT, et al. Preserved T-cell function in children and young adults with immune-tolerant chronic hepatitis B. Gastroenterology. 2012;143(3):637-45.
6. Tang ZS, Hao YH, Zhang EJ, Xu CL, Zhou Y, Zheng X, et al. CD28 family of receptors on T cells in chronic HBV infection: Expression characteristics, clinical significance and correlations with PD-1 blockade. Mol Med Rep. 2016;14(2):1107-16.
7. Pang X, Zhang L, Liu N, Liu B, Chen Z, Li H, et al. Combination of pegylated interferon-alpha and nucleos(t)ide analogue treatment enhances the activity of natural killer cells in nucleos(t)ide analogue experienced chronic hepatitis B patients. Clin Exp Immunol. 2020;202(1):80-92.
8. Raziorrouh B, Heeg M, Kurktschiev P, Schraut W, Zachoval R, Wendtner C, et al. Inhibitory Phenotype of HBV-Specific CD4+ T-Cells Is Characterized by High PD-1 Expression but Absent Coregulation of Multiple Inhibitory Molecules. PLoS ONE. 2014;9(8):e105703.
9. Wang X, He Q, Shen H, Lu X, Sun B. Genetic and phenotypic difference in CD8+ T cell exhaustion between chronic hepatitis B infection and hepatocellular carcinoma. J Med Genet. 2019:18-21.
10. Schurich A, Khanna P, Lopes AR, Han KJ, Peppa D, Micco L, et al. Role of the coinhibitory receptor cytotoxic T lymphocyte antigen-4 on apoptosis-Prone CD8 T cells in persistent hepatitis B virus infection. Hepatology. 2011;53(5):1494-503.
11. Fisicaro P, Valdatta C, Massari M, Loggi E, Ravanetti L, Urbani S, et al. Combined blockade of programmed death-1 and activation of CD137 increase responses of human liver T cells against HBV, but not HCV. Gastroenterology. 2012;143(6):1576-85 e4.
12. Bengsch B, Martin B, Thimme R. Restoration of HBV-specific CD8+ T cell function by PD-1 blockade in inactive carrier patients is linked to T cell differentiation. J Hepatol. 2014;61(6):1212-9.
13. Pal S, Dey D, Chakraborty BC, Nandi M, Khatun M, Banerjee S, et al. Diverse facets of MDSC in different phases of chronic HBV infection: Impact on HBV-specific T-cell response and homing. Hepatology. 2022;76(3):759-74.
14. Liu Y, Cheng L-s, Wu S-d, Wang S-q, Li L, She W-m, et al. IL-10-producing regulatory B-cells suppressed effector T-cells but enhanced regulatory T-cells in chronic HBV infection. Clinical Science. 2016;130(11):907-19.
15. Stoop JN, Claassen MA, Woltman AM, Binda RS, Kuipers EJ, Janssen HL, et al. Intrahepatic regulatory T cells are phenotypically distinct from their peripheral counterparts in chronic HBV patients. Clin Immunol. 2008;129(3):419-27.
16. Kondo Y, Kobayashi K, Ueno Y, Shiina M, Niitsuma H, Kanno N, et al. Mechanism of T cell hyporesponsiveness to HBcAg is associated with regulatory T cells in chronic hepatitis B. World J Gastroenterol. 2006;12(27):4310-7.

17. Cao W, Qiu ZF, Li TS. Parallel decline of CD8+CD38+ lymphocytes and viremia in treated hepatitis B patients. World J Gastroenterol. 2011;17(17):2191-8.
18. Shen XH, Xu P, Yu X, Song HF, Chen H, Zhang XG, et al. Discrepant Clinical Significance of CD28(+)CD8(-) and CD4(+)CD25(high) Regulatory T Cells During the Progression of Hepatitis B Virus Infection. Viral Immunol. 2018;31(8):548-58.
19. Li X, Kong H, Tian L, Zhu Q, Wang Y, Dong Y, et al. Changes of costimulatory molecule CD28 on circulating CD8+ T cells correlate with disease pathogenesis of chronic hepatitis B. Biomed Res Int. 2014;2014:423181.
20. Jiao L, Chen J, Wu X, Cai B, Su Z, Wang L. Correlation of CpG methylation of the Pdcd1 gene with PD-1 expression on CD8(+) T cells and medical laboratory indicators in chronic hepatitis B infection. J Gene Med. 2020;22(2):e3148.
21. Yang Z, Lei Y, Chen C, Ren H, Shi T. Roles of the programmed cell death 1, T cell immunoglobulin mucin-3, and cluster of differentiation 288 pathways in the low reactivity of invariant natural killer T cells after chronic hepatitis B virus infection. Arch Virol. 2015;160(10):2535-45.
22. Lu GF, Tang FA, Zheng PY, Yang PC, Qi YM. Entecavir up-regulates dendritic cell function in patients with chronic hepatitis B. World J Gastroenterol. 2008;14(10):1617-21.
23. Wang F, Xing L, Liu M, Zhu C, Liu H, Wang H, et al. Dysfunction of peripheral blood dendritic cells from patients with chronic hepatitis B virus infection. World J Gastroenterol. 2001;7(4):537–41.
24. Li R, Chen H, Xie Y, Fei R, Cong X, Jiang D, et al. Dendritic cells from chronic hepatitis B patients can induce HBV antigen-specific T cell responses. World J Gastroenterol. 2004;10(11):1578–82.
25. Lin C, Zou H, Wang S. Hepatitis B e Antigen Seroconversion Is Related with the Function of Dendritic Cells in Chronic Hepatitis B Virus Infection. Gastroenterol Res Pract. 2014;2014:413952.
26. Yu Y, Tang Z, Han J, Xi M, Feng J, Zang G. Expression of ICAM-1, HLA-DR, and CD80 on peripheral circulating CD1α DCs induced in vivo by IFN- α in patients with chronic hepatitis B. World J Gastroenterol. 2006;12(9):1447–51.
27. Tjwa ET, van Oord GW, Biesta PJ, Boonstra A, Janssen HL, Woltman AM. Restoration of TLR3-activated myeloid dendritic cell activity leads to improved natural killer cell function in chronic hepatitis B virus infection. J Virol. 2012;86(8):4102-9.
28. Duan X, Zhuang H, Wang M, Li H, Liu J, Wang F. Decreased numbers and impaired function of circulating dendritic cell subsets in patients with chronic hepatitis B infection (R2). J Gastroenterol Hepatol. 2005;March 2004:234–42.
29. Chen L, Zhang Z, Chen W, Zhang Z, Li Y, Shi M, et al. B7-H1 Up-Regulation on Myeloid Dendritic Cells Significantly Suppresses T Cell Immune Function in Patients with Chronic Hepatitis B. J Immunol. 2007.
30. Tavakoli S, Mederacke I, Herzog-Hauff S, Glebe D, Grün S, Strand D, et al. Peripheral blood dendritic cells are phenotypically and functionally intact in chronic hepatitis B virus (HBV) infection. Clinical and Experimental Immunology. 2007;151(1):61-70.
31. van der Molen RG, Sprengers D, Binda RS, de Jong EC, Niesters HG, Kusters JG, et al. Functional impairment of myeloid and plasmacytoid dendritic cells of patients with chronic hepatitis B. Hepatology. 2004;40(3):738-46.
32. Pan X, Yao W, Fu J, Liu M, Li L, Gao X. Telbivudine improves the function of myeloid dendritic cells in patients with chronic hepatitis B. Acta virologica. 2012;56(1):31-8.
33. Said EA, Al-Reesi I, Al-Riyami M, Al-Naamani K, Al-Sinawi S, Al-Balushi MS, et al. Increased CD86 but Not CD80 and PD-L1 Expression on Liver CD68+ Cells during Chronic HBV Infection. PLoS One. 2016;11(6):e0158265.
34. Xu X, Shang Q, Chen X, Nie W, Zou Z, Huang A, et al. Reversal of B-cell hyperactivation and functional impairment is associated with HBsAg seroconversion in chronic hepatitis B patients. Cellular & Molecular Immunology. 2015;12(3):309-16.

35. Peng M, Chen M, Ling N, Xu H, Qing Y, Ren H. Novel vaccines for the treatment of chronic HBV infection based on mycobacterial heat shock protein 70. Vaccine. 2006;24(7):887-96.
36. Xiang XX, Zhou XQ, Wang JX, Xie Q, Cai X, Yu H, et al. Effects of CpG-ODNs on phenotype and function of monocyte-derived dendritic cells in chronic hepatitis B. World J Gastroenterol. 2011;17(43):4825-30.
37. Kunitani H, Shimizu Y, Murata H, Higuchi K, Watanabe A. Phenotypic analysis of circulating and intrahepatic dendritic cell subsets in patients with chronic liver diseases. Journal of Hepatology. 2002;32:734–41.
38. Martinet J, Dufeu–Duchesne T, Bruder Costa J, Larrat S, Marlu A, Leroy V, et al. Altered Functions of Plasmacytoid Dendritic Cells and Reduced Cytolytic Activity of Natural Killer Cells in Patients With Chronic HBV Infection. Gastroenterology. 2012;143(6):1586-96.e8.
39. Li M-H, Zhang L, Zhang D, Cao W-H, Qi T-L, Hao H-X, et al. Plasmacytoid Dendritic Cell Function and Cytokine Network Profiles in Patients with Acute or Chronic Hepatitis B Virus Infection. Chinese Medical Journal. 2018;131(01):43-9.
40. Wang K, Pei H, Huang B, Yang RL, Wu HY, Zhu X, et al. Overexpression of Fc receptor-like 1 associated with B-cell activation during hepatitis B virus infection. Braz J Med Biol Res. 2012;45(12):1112-8.
41. Oliviero B, Cerino A, Varchetta S, Paudice E, Pai S, Ludovisi S. Enhanced B-cell differentiation and reduced proliferative capacity in chronic hepatitis C and chronic hepatitis B virus infections. J Hepatol. 2011;55(1):53-60.
42. Cai G, Nie X, Li L, Hu L, Wu B, Lin J, et al. B and T lymphocyte attenuator is highly expressed on intrahepatic T cells during chronic HBV infection and regulates their function. Journal of Gastroenterology. 2013;48(12):1362-72.
43. Hou F-Q, Wu X-J, Wang Y, Chen J, Liu Y-Z, Ren Y-Y, et al. Rapid downregulation of programmed death-1 and interferon-γ-inducible protein-10 expression is associated with favourable outcome during antiviral treatment of chronic hepatitis B. Journal of Viral Hepatitis. 2013;20(s1):18-26.
44. Li CZ, Hu JJ, Xue JY, Yin W, Liu YY, Fan WH, et al. Viral infection parameters not nucleoside analogue itself correlates with host immunity in nucleoside analogue therapy for chronic hepatitis B. World J Gastroenterol. 2014;20(28):9486-96.
45. Xu P, Chen YJ, Chen H, Zhu XY, Song HF, Cao LJ, et al. The expression of programmed death-1 in circulating CD4+ and CD8+ T cells during hepatitis B virus infection progression and its correlation with clinical baseline characteristics. Gut and liver. 2014;8(2):186-95.
46. Nan X-P, Zhang Y, Yu H-T, Li Y, Sun R-L, Wang J-P, et al. Circulating CD4+CD25high Regulatory T Cells and Expression of PD-1 and BTLA on CD4+ T Cells in Patients with Chronic Hepatitis B Virus Infection. Viral Immunology. 2010;23(1):63-70.
47. Li M, Sun XH, Zhu XJ, Jin SG, Zeng ZJ, Zhou ZH, et al. HBcAg induces PD-1 upregulation on CD4+T cells through activation of JNK, ERK and PI3K/AKT pathways in chronic hepatitis-B-infected patients. Laboratory investigation; a journal of technical methods and pathology. 2012;92(2):295-304.
48. Rinker F, Zimmer CL, Höner Zu Siederdissen C, Manns MP, Kraft ARM, Wedemeyer H, et al. Hepatitis B virus-specific T cell responses after stopping nucleos(t)ide analogue therapy in HBeAg-negative chronic hepatitis B. J Hepatol. 2018;69(3):584-93.
49. Liu W, Zheng X, Wang J, He Q, Li J, Zhang Z, et al. MicroRNA-138 Regulates T-Cell Function by Targeting PD-1 in Patients with Hepatitis B Virus-Related Liver Diseases. Lab Med. 2021;52(5):439-51.
50. Cooksley H, Riva A, Katzarov K, Hadzhiolova-Lebeau T, Pavlova S, Simonova M, et al. Differential Expression of Immune Inhibitory Checkpoint Signatures on Antiviral and Inflammatory T Cell Populations in Chronic Hepatitis B. Journal of Interferon & Cytokine Research. 2018;38(7):273-82.

51. Dong Y, Li X, Zhang L, Zhu Q, Chen C, Bao J, et al. CD4(+) T cell exhaustion revealed by high PD-1 and LAG-3 expression and the loss of helper T cell function in chronic hepatitis B. BMC Immunol. 2019;20(1):27.
52. Jacobi FJ, Wild K, Smits M, Zoldan K, Csernalabics B, Flecken T, et al. OX40 stimulation and PD-L1 blockade synergistically augment HBV-specific CD4 T cells in patients with HBeAg-negative infection. J Hepatol. 2019;70(6):1103-13.
53. Nan XP, Zhang Y, Yu HT, Sun RL, Peng MJ, Li Y, et al. Inhibition of viral replication downregulates CD4(+)CD25(high) regulatory T cells and programmed death-ligand 1 in chronic hepatitis B. Viral Immunol. 2012;25(1):21-8.
54. Ma SW, Huang X, Li YY, Tang LB, Sun XF, Jiang XT, et al. High serum IL-21 levels after 12 weeks of antiviral therapy predict HBeAg seroconversion in chronic hepatitis B. J Hepatol. 2012;56(4):775-81.
55. Ji LS, Gao QT, Guo RW, Zhang X, Zhou ZH, Yu Z, et al. Immunomodulatory Effects of Combination Therapy with Bushen Formula plus Entecavir for Chronic Hepatitis B Patients. J Immunol Res. 2019;2019:8983903.
56. Peng G, Li S, Wu W, Tan X, Chen Y, Chen Z. PD-1 upregulation is associated with HBV-specific T cell dysfunction in chronic hepatitis B patients. Molecular immunology. 2008;45(4):963-70.
57. Ye P, Weng ZH, Zhang SL, Zhang JA, Zhao L, Dong JH, et al. Programmed death-1 expression is associated with the disease status in hepatitis B virus infection. World J Gastroenterol. 2008;14(28):4551-7.
58. Liang XS, Zhou Y, Li CZ, Wan MB. Natural course of chronic hepatitis B is characterized by changing patterns of programmed death type-1 of CD8-positive T cells. World J Gastroenterol. 2010;16(5):618-24.
59. Diao B, Huang X, Guo S, Yang C, Liu G, Chen Y, et al. MAGT1-mediated disturbance of Mg(2+) homeostasis lead to exhausted of HBV-infected NK and CD8(+) T cells. Sci Rep. 2017;7(1):13594.
60. Ma L, Cai YJ, Yu L, Feng JY, Wang J, Li C, et al. Treatment with telbivudine positively regulates antiviral immune profiles in Chinese patients with chronic hepatitis B. Antimicrobial agents and chemotherapy. 2013;57(3):1304-11.
61. Sung PS, Park DJ, Kim JH, Han JW, Lee EB, Lee GW, et al. Ex vivo Detection and Characterization of Hepatitis B Virus-Specific CD8(+) T Cells in Patients Considered Immune Tolerant. Front Immunol. 2019;10:1319.
62. de Niet A, de Bruijne J, Plat-Sinnige MJ, Takkenberg RB, van Lier RA, Reesink HW, et al. Upregulation of CXCR3 expression on CD8+ T cells due to the pervasive influence of chronic hepatitis B and C virus infection. Human immunology. 2013;74(8):899-906.
63. Fisicaro P, Valdatta C, Massari M, Loggi E, Biasini E, Sacchelli L, et al. Antiviral intrahepatic T-cell responses can be restored by blocking programmed death-1 pathway in chronic hepatitis B. Gastroenterology. 2010;138(2):682-93, 93 e1-4.
64. Xibing G, Xiaojuan Y, Juanhua W. PD-1 expression on CTL may be related to more severe liver damage in CHB patients with HBV genotype C than in those with genotype B infection. J Viral Hepat. 2013;20(4):e1-2.
65. Aoki J, Kowazaki Y, Ohtsuki T, Okamoto R, Ohashi K, Hayashi S, et al. Kinetics of peripheral hepatitis B virus-specific CD8+ T cells in patients with onset of viral reactivation. J Gastroenterol. 2013;48(6):728-37.
66. Fan R, Lan Y, Chen J, Huang Y, Yan Q, Jiang L, et al. T-bet expression in CD8+ T cells associated with chronic hepatitis B virus infection. Virology journal. 2016;13:14.
67. Zhang WJ, Peng CH, Zheng SS. Programmed death 1 and programmed death ligand 1 expressions in patients with chronic hepatitis B. Hepatobiliary & pancreatic diseases international : HBPD INT. 2013;12(4):394-9.

68. Ferrando-Martinez S, Snell Bennett A, Lino E, Gehring AJ, Feld J, Janssen HLA, et al. Functional Exhaustion of HBV-Specific CD8 T Cells Impedes PD-L1 Blockade Efficacy in Chronic HBV Infection. Front Immunol. 2021;12:648420.
69. Wenjin Z, Chuanhui P, Yunle W, Lateef SA, Shusen Z. Longitudinal fluctuations in PD1 and PD-L1 expression in association with changes in anti-viral immune response in chronic hepatitis B. BMC gastroenterology. 2012;12:109.
70. Evans A, Riva A, Cooksley H, Phillips S, Puranik S, Nathwani A, et al. Programmed death 1 expression during antiviral treatment of chronic hepatitis B: Impact of hepatitis B e-antigen seroconversion. Hepatology. 2008;48(3):759-69.
71. Mohammadizad H, Shahbazi M, Hasanjani Roushan MR, Soltanzadeh-Yamchi M, Mohammadnia-Afrouzi M. TIM-3 as a marker of exhaustion in CD8(+) T cells of active chronic hepatitis B patients. Microbial pathogenesis. 2019;128:323-8.
72. TrehanPati N, Kotillil S, Hissar SS, Shrivastava S, Khanam A, Sukriti S, et al. Circulating Tregs correlate with viral load reduction in chronic HBV-treated patients with tenofovir disoproxil fumarate. J Clin Immunol. 2011;31(3):509-20.
73. Liu YZ, Hou FQ, Ding P, Ren YY, Li SH, Wang GQ. Pegylated interferon α enhances recovery of memory T cells in e antigen positive chronic hepatitis B patients. Virology journal. 2012;9:274.
74. Gu XB, Yang XJ, Hua Z, Lu ZH, Zhang B, Zhu YF, et al. Effect of oxymatrine on specific cytotoxic T lymphocyte surface programmed death receptor-1 expression in patients with chronic hepatitis B. Chin Med J (Engl). 2012;125(8):1434-8.
75. Schuch A, Alizei ES, Heim K, Wieland D, Kiraithe MM, Kemming J, et al. Phenotypic and functional differences of HBV core-specific versus HBV polymerase-specific CD8+ T cells in chronically HBV-infected patients with low viral load. Gut. 2019;68(5):905-15.
76. Dinney CM, Zhao LD, Conrad CD, Duker JM, Karas RO, Hu Z, et al. Regulation of HBV-specific CD8(+) T cell-mediated inflammation is diversified in different clinical presentations of HBV infection. Journal of microbiology (Seoul, Korea). 2015;53(10):718-24.
77. Boni C, Fisicaro P, Valdatta C, Amadei B, Di Vincenzo P, Giuberti T, et al. Characterization of hepatitis B virus (HBV)-specific T-cell dysfunction in chronic HBV infection. J Virol. 2007;81(8):4215-25.
78. Gehring AJ, Ho ZZ, Tan AT, Aung MO, Lee KH, Tan KC, et al. Profile of tumor antigen-specific CD8 T cells in patients with hepatitis B virus-related hepatocellular carcinoma. Gastroenterology. 2009;137(2):682-90.
79. Xu Z, Lin JZ, Zeng YF, Yang XH, Wu ZB, Hu ZX, et al. Changes of cytokine levels and T cell surface molecules in patients with chronic hepatitis B and the association with functional cure. J Med Virol. 2021;93(8):4966-74.
80. Chen J, Wang XM, Wu XJ, Wang Y, Zhao H, Shen B, et al. Intrahepatic levels of PD-1/PD-L correlate with liver inflammation in chronic hepatitis B. Inflamm Res. 2011;60(1):47-53.
81. Huang ZY, Xu P, Li JH, Zeng CH, Song HF, Chen H, et al. Clinical Significance of Dynamics of Programmed Death Ligand-1 Expression on Circulating CD14(+) Monocytes and CD19(+) B Cells with the Progression of Hepatitis B Virus Infection. Viral Immunol. 2017;30(3):224-31.
82. Huang A, Zhang B, Yan W, Wang B, Wei H, Zhang F, et al. Myeloid-derived suppressor cells regulate immune response in patients with chronic hepatitis B virus infection through PD-1-induced IL-10. J Immunol. 2014;193(11):5461-9.
83. Feng C, Cao LJ, Song HF, Xu P, Chen H, Xu JC, et al. Expression of PD-L1 on CD4+CD25+Foxp3+ Regulatory T Cells of Patients with Chronic HBV Infection and Its Correlation with Clinical Parameters. Viral Immunol. 2015;28(8):418-24.
84. Sharma S, Khosla R, David P, Rastogi A, Vyas A, Singh D, et al. CD4+CD25+CD127(low) Regulatory T Cells Play Predominant Anti-Tumor Suppressive Role in Hepatitis B Virus-Associated Hepatocellular Carcinoma. Front Immunol. 2015;6:49.

85. Li H, Zhai N, Wang. Z, Song H, Yang Y, Cui A, et al. Regulatory NK cells mediated between immunosuppressive monocytes and dysfunctional T cells in chronic HBV infection. Gut. 2018;67:2035-44.
86. Li X, Zhou L, Gu L, Gu Y, Chen L, Lian Y, et al. Veritable antiviral capacity of natural killer cells in chronic HBV infection: an argument for an earlier anti-virus treatment. Journal of translational medicine. 2017;15(1):220.
87. Huang X, Mo Q, Fu T, Liu Y, Diao B. STAT1 is associated with NK cell dysfunction by downregulating NKG2D transcription in chronic HBV-infected patients. Immunobiology. 2022;227(6):152272.
88. Shi TD, Zhang JM, Wang XF, Chen M, Sun H, Chen CB, et al. Effects of antiviral therapy with Telbivudine on peripheral iNKT cells in HBeAg(+) chronic hepatitis B patients. Clinical and experimental medicine. 2012;12(2):105-13.
89. Han Y, Li J, Jiang L, Xu Q, Liu B, Jin K, et al. Regulation of B7-H1 expression on peripheral monocytes and IFN-γ secretion in T lymphocytes by HBeAg. Cell Immunol. 2013;283(1-2):25-30.
90. Liu N, Liu B, Zhang L, Li H, Chen Z, Luo A, et al. Recovery of circulating CD56(dim) NK cells and the balance of Th17/Treg after nucleoside analog therapy in patients with chronic hepatitis B and low levels of HBsAg. International immunopharmacology. 2018;62:59-66.
91. Ye B, Li X, Dong Y, Wang Y, Tian L, Lin S, et al. Increasing LAG-3 expression suppresses T-cell function in chronic hepatitis B: A balance between immunity strength and liver injury extent. Medicine. 2017;96(1):e5275.
92. Xie C, Wang S, Zhang H, Zhu Y, Jiang P, Shi S, et al. Lnc-AIFM2-1 promotes HBV immune escape by acting as a ceRNA for miR-330-3p to regulate CD244 expression. Front Immunol. 2023;14:1121795.
93. Raziorrouh B, Schraut W, Gerlach T, Nowack D, Grüner NH, Ulsenheimer A, et al. The immunoregulatory role of CD244 in chronic hepatitis B infection and its inhibitory potential on virus-specific CD8+ T-cell function. Hepatology. 2010;52(6):1934-47.
94. Zhang L, Wang Q, Zhao P, Hu X, Jiang Y. Effects of entecavir on peripheral blood lymphocyte profiles in chronic hepatitis B patients with suboptimal responses to adefovir. Clin Exp Pharmacol Physiol. 2014;41(7):514-23.
95. Scott-Algara D, Mancini-Bourgine M, Fontaine H, Pol S, Michel ML. Changes to the natural killer cell repertoire after therapeutic hepatitis B DNA vaccination. PLoS One. 2010;5(1):e8761.
96. Sun C, Fu B, Gao Y, Liao X, Sun R, Tian Z, et al. TGF-β1 down-regulation of NKG2D/DAP10 and 2B4/SAP expression on human NK cells contributes to HBV persistence. PLoS Pathog. 2012;8(3):e1002594.
97. Wijaya RS, Read SA, Schibeci S, Eslam M, Azardaryany MK, El-Khobar K, et al. KLRG1+ natural killer cells exert a novel antifibrotic function in chronic hepatitis B. J Hepatol. 2019;71(2):252-64.
98. Zong L, Peng H, Sun C, Li F, Zheng M, Chen Y, et al. Breakdown of adaptive immunotolerance induces hepatocellular carcinoma in HBsAg-tg mice. Nature Communications. 2019;10(1):221.
99. Liu S, Xu C, Yang F, Zong L, Qin Y, Gao Y, et al. Natural Killer Cells Induce CD8(+) T Cell Dysfunction via Galectin-9/TIM-3 in Chronic Hepatitis B Virus Infection. Front Immunol. 2022;13:884290.
100. Song HF, Chen XJ, Tang PJ, Xu P, Huang ZY, Wang XF. Clinical Significance of BTLA and HVEM Expression on Circulating CD4(+) T and CD8(+) T Cells in Chronic Hepatitis B Virus Infection. Viral Immunol. 2022;35(4):291-302.
101. Wang H, Wu B, Li L, Hu L, Lin J, Jiang C, et al. Hepatic expansion of virus-specific CD8(+)BTLA(+) T cells with regulatory properties in chronic hepatitis B virus infection. Cell Immunol. 2017;311:36-45.

102. Nebbia G, Peppa D, Schurich A, Khanna P, Singh HD, Cheng Y, et al. Upregulation of the Tim-3/galectin-9 pathway of T cell exhaustion in chronic hepatitis B virus infection. PLoS One. 2012;7(10):e47648.
103. Dong J, Yang XF, Wang LX, Wei X, Wang AH, Hao CQ, et al. Modulation of Tim-3 Expression by Antigen-Dependent and -Independent Factors on T Cells from Patients with Chronic Hepatitis B Virus Infection. Frontiers in cellular and infection microbiology. 2017;7:98.
104. Wu W, Shi Y, Li S, Zhang Y, Liu Y, Wu Y, et al. Blockade of Tim-3 signaling restores the virus-specific $CD8^+$ T-cell response in patients with chronic hepatitis B. European journal of immunology. 2012;42(5):1180-91.
105. Wu W, Shi Y, Li J, Chen F, Chen Z, Zheng M. Tim-3 expression on peripheral T cell subsets correlates with disease progression in hepatitis B infection. Virology journal. 2011;8:113.
106. Wang J, Li C, Fu J, Wang X, Feng X, Pan X. Tim-3 regulates inflammatory cytokine expression and Th17 cell response induced by monocytes from patients with chronic hepatitis B. Scandinavian journal of immunology. 2019;89(5):e12755.
107. Rong YH, Wan ZH, Song H, Li YL, Zhu B, Zang H, et al. Tim-3 expression on peripheral monocytes and CD3+CD16/CD56+natural killer-like T cells in patients with chronic hepatitis B. Tissue antigens. 2014;83(2):76-81.
108. Hu CC, Jeng WJ, Chen YC, Fang JH, Huang CH, Teng W, et al. Memory Regulatory T cells Increase Only In Inflammatory Phase of Chronic Hepatitis B Infection and Related to Galectin-9/Tim-3 interaction. Sci Rep. 2017;7(1):15280.
109. Ju Y, Hou N, Meng J, Wang X, Zhang X, Zhao D, et al. T cell immunoglobulin- and mucin-domain-containing molecule-3 (Tim-3) mediates natural killer cell suppression in chronic hepatitis B. J Hepatol. 2010;52(3):322-9.
110. Norouzi A, Taziki S, Najafipasandi A, Mohammadi S, Roshandel G. Rosuvastatin Intervention Decreased the Frequencies of the TIM-3+ Population of NK Cells and NKT Cells among Patients with Chronic Hepatitis B. Iran J Immunol. 2022;19(3):255-62.
111. Lv G, Ying L, Ma WJ, Jin X, Zheng L, Li L, et al. Dynamic analysis of CD127 expression on memory CD8 T cells from patients with chronic hepatitis B during telbivudine treatment. Virology journal. 2010;7:207.
112. Tavakoli S, Schwerin W, Rohwer A, Hoffmann S, Weyer S, Weth R, et al. Phenotype and function of monocyte derived dendritic cells in chronic hepatitis B virus infection. The Journal of general virology. 2004;85(Pt 10):2829-36.
113. Xing T, Xu H, Yu W. Role of T follicular helper cells and their associated molecules in the pathogenesis of chronic hepatitis B virus infection. Exp Ther Med. 2013;5(3):885-9.
114. Barboza L, Salmen S, Peterson DL, Montes H, Colmenares M, Hernández M, et al. Altered T cell costimulation during chronic hepatitis B infection. Cell Immunol. 2009;257(1-2):61-8.
115. Toubi E, Kessel A, Goldstein L, Slobodin G, Sabo E, Shmuel Z, et al. Enhanced peripheral T-cell apoptosis in chronic hepatitis C virus infection: association with liver disease severity. J Hepatol. 2001;35(6):774-80.
116. Liu Y, Wang G, Chen Y, Huang R, Tian C, Li Y, et al. HBcAg-induced upregulated 4-1BB ligand on B cells contributes to B-cell hyperactivation during chronic hepatitis B infection. Journal of Medical Virology. 2019;91(5):781-90.
117. Publicover J, Gaggar A, Jespersen JM, Halac U, Johnson AJ, Goodsell A, et al. An OX40/OX40L interaction directs successful immunity to hepatitis B virus. Science translational medicine. 2018;10(433).
